# Comparative Transcriptome Analysis of Shoots and Roots of TNG67 and TCN1 Rice Seedlings under Cold Stress and Following Subsequent Recovery: Insights into Metabolic Pathways, Phytohormones, and Transcription Factors

**DOI:** 10.1371/journal.pone.0131391

**Published:** 2015-07-02

**Authors:** Yun-Wei Yang, Hung-Chi Chen, Wei-Fu Jen, Li-Yu Liu, Men-Chi Chang

**Affiliations:** Department of Agronomy, National Taiwan University, No. 1, Section 4, Taipei, Taiwan; CSIR-National Botanical Research Institute, INDIA

## Abstract

Cold stress affects rice growth, quality and yield. The investigation of genome-wide gene expression is important for understanding cold stress tolerance in rice. We performed comparative transcriptome analysis of the shoots and roots of 2 rice seedlings (TNG67, cold-tolerant; and TCN1, cold-sensitive) in response to low temperatures and restoration of normal temperatures following cold exposure. TNG67 tolerated cold stress via rapid alterations in gene expression and the re-establishment of homeostasis, whereas the opposite was observed in TCN1, especially after subsequent recovery. Gene ontology and pathway analyses revealed that cold stress substantially regulated the expression of genes involved in protein metabolism, modification, translation, stress responses, and cell death. TNG67 takes advantage of energy-saving and recycling resources to more efficiently synthesize metabolites compared with TCN1 during adjustment to cold stress. During recovery, expression of OsRR4 type-A response regulators was upregulated in TNG67 shoots, whereas that of genes involved in oxidative stress, chemical stimuli and carbohydrate metabolic processes was downregulated in TCN1. Expression of genes related to protein metabolism, modification, folding and defense responses was upregulated in TNG67 but not in TCN1 roots. In addition, abscisic acid (ABA)-, polyamine-, auxin- and jasmonic acid (JA)-related genes were preferentially regulated in TNG67 shoots and roots and were closely associated with cold stress tolerance. The TFs AP2/ERF were predominantly expressed in the shoots and roots of both TNG67 and TCN1. The TNG67-preferred TFs which express in shoot or root, such as OsIAA23, SNAC2, OsWRKY1v2, 24, 53, 71, HMGB, OsbHLH and OsMyb, may be good candidates for cold stress tolerance-related genes in rice. Our findings highlight important alterations in the expression of cold-tolerant genes, metabolic pathways, and hormone-related and TF-encoding genes in TNG67 rice during cold stress and recovery. The cross-talk of hormones may play an essential role in the ability of rice plants to cope with cold stress.

## Introduction

Rice is the most important staple food in the world, especially in Asia. Two subspecies of rice, *Oryza sativa* ssp. *japonica* (temperate rice) and ssp. *indica* (tropical rice), are widely grown in different areas. Rice seedlings frequently experience cold injury, which affects their growth and yield. In general, *indica* rice tends to be more sensitive to low temperatures. Thus, to further improve rice quality and production and to overcome the limiting factor of cold, a thorough understanding of cold stress tolerance mechanisms in rice is needed, especially the differential means of cold stress perception and responses to this type of stress in the *indica* (e.g., TCN1) and *japonica* (e.g., TNG67) rice varieties.

To adapt to environmental stresses, energy conservation and metabolic homeostasis are pivotal for all organisms. Under cold stress, various physiological and biochemical responses are altered in plants, such as the inhibition of photosynthesis, respiration and protein translation, accumulation of reactive oxygen species (ROS), alterations in metabolite profiles and osmolyte adjustment. Therefore, energy deprivation is likely a consequence of stress damage, which is often associated with reduced photosynthesis or respiration, ultimately resulting in growth arrest and cell death. Under abiotic stress, plants can reprogram or reconfigure their primary metabolism to redistribute energy resources for survival [1]. Alterations in primary metabolism involving sugars and sugar alcohols, amino acids and tricarboxylic acid (TCA) cycle intermediates are general trends in abiotic stress responses [2].

Many growth and developmental processes in plants are affected by the balance and coordination of different plant hormones. Fluctuations in stress-responsive phytohormone levels are central to integrating stress signaling and regulating stress responses [3]. However, the involvement of plant hormones in abiotic stress, especially cold stress tolerance, in rice remains poorly understood. To reveal the activities of plant hormones in rice seedlings at low temperatures, cold damage and levels of plant hormones, including abscisic acid (ABA), ethylene (ET) and polyamine, were evaluated in seedlings of 2 rice cultivars with contrasting responses to cold, TNG67 and TCN1 [4]. The TNG67 seedlings were remarkably cold-tolerant compared with those of TCN1, as reflected by electrolyte leakage, the tetrazolium chloride reduction assay results and the survival ratio. After incubation at 5°C for 3 hr, the stomata of TNG67 immediately closed, but those of TCN1 did not, indicating the presence of wilt symptoms in TCN1 [5]. In the cold-tolerant cultivar TNG67, ABA levels rapidly increased in roots and shoots in response to cold stress, but this did not occur in the cold-sensitive cultivar TCN1. Interestingly, exogenous addition of ABA enhanced cold stress resistance in TCN1 rice seedlings. The levels of both 1-amino-cyclopropane-1-carboxylic acid (ACC) and ET were decreased in TNG67 and TCN1 in response to cold treatment, and TCN1 was not able to restore the ET level after removal from the cold [4]. The level of another plant hormone, putrescine (Put), and the activity of arginine decarboxylase (ADC) were enhanced in both the shoots and roots of TNG67. The levels of spermidine/spermine and activity of S-adenosylmethionine decarboxylase (SAMDC) were increased in TNG67 shoots. In TCN1, although the level of Put and activity of ADC were increased in shoots, the magnitudes of these increases were smaller than those observed in TNG67. Further, the Put level in roots was decreased after cold treatment [6]. Moreover, the treatment of whole rice plants with 10 mM methyl jasmonate (MeJA) for 48 hr prior to the cold treatment induced cold tolerance in TCN1 [7].

Cold stress induces global gene expression changes in rice. Many transcription factors (TFs) are involved in the gene regulatory networks (regulons) of plant cold stress tolerance. In plants, C-repeat binding factor (CRB), also known as dehydration-responsive element-binding protein (DREB) regulon, is involved in the most well-known mechanism associated with cold stress. During cold acclimation, INDUCER OF C-REPEAT BINDING FACTOR (CBF) EXPRESSION 1 (*ICE*), a MYC-type basic helix–loop–helix TF, binds to the *CBF* promoter to regulate its gene expression. Cold-induced CBFs, which are members of the APETALA2/ETHYLENE RESPONSE FACTOR (AP2/ERF) gene family, can induce cold response (*COR*) genes by binding to the *cis*-acting elements in their promoters [8]. In addition, other rice TFs that are involved in cold acclimation is induced under these conditions. For example, the overexpression of rice MYB4 (an R2R3-type MYB) [9] and OsMYB3R-2 (an R1R2R3 MYB) [10] enhances tolerance to freezing in *Arabidopsis*. The overexpression of low-temperature–induced *OsSNAC2* increases cell membrane stability under cold stress [11].

Recently, rice microarrays with accurate gene coverage have been widely used to obtain a better understanding of global changes in gene expression profiles in response to cold stress. [12]. To date, there is a lack of detailed knowledge regarding subspecies-, tissue- and temporal-specific gene expression changes, as well as metabolic or gene-regulatory network remodeling, during cold stress and subsequent recovery in rice. In particular, little is known about the gene expression and pathway changes related to hormone homeostasis under cold stress. Thus, to identify favorable alleles in *indica* that may increase the cold tolerance of *japonica* rice, Zhang et al. [13] have used K354 and C418, a cold-tolerant introgression line and its corresponding cold-sensitive recurrent parent rice, respectively, for genome-wide expression analyses. More than 195 TFs were found, and half of the differentially expressed genes (DEGs) identified were associated with the *OsDREB1* and *OsMyb4* regulons. The genes that were specifically induced under cold stress in K354 functioned in cell wall organization, microtubule-based movement and defense. Candidate genes in introgression segments, such as *OsWAK112d*, *OsFAD7*, *Sir2* and programmed cell death (PCD)-associated genes, may be responsible for cold tolerance in rice. Zhang et al. [14] have conducted comparative transcriptome analyses of the shoots of rice cultivars with contrasting responses to cold, LTH and IR29, and have shown that the ability to recover after chilling stress may be an important factor in rice cold stress tolerance. DEG and *cis*-acting element analyses have indicated that the *CBF* and *MYBS3* regulons are associated with resistance to low temperatures in rice. A number of genes have been shown to co-localize with 5 cold tolerance quantitative trait loci, indicating that they are candidate genes for cold tolerance in rice. Chawade et al. [15] have used a specific cold-tolerant rice variety (Jumli Marshi, a Nepalese highland *japonica* variety) in comparative microarray data analysis. Promoter analysis of the DEGs identified has revealed complicated cross-talk between the CBF and ABRE regulons and a role of ABA signaling in rice cold tolerance. Other microarray studies have indicated that the early regulatory response can be triggered by oxidative signals in response to low temperatures [16, 17].

The majority of the above-mentioned research has focused on identifying genes and TFs that participate in cold-tolerant mechanisms. However, because of the different genotypes, tissues, growth stages and treatment conditions assessed, comparisons of these microarray results may not reveal similarities among them or allow for the identification of the manner by which temperature affects root water uptake and hydraulic conductivity and reduces cold tolerance. In chilling-resistant species, hydraulic conductivity increases at low temperatures to prevent cold-induced water stress [18]. The cold acclimation process is improved in rice by cold tolerance-enhanced water uptake by roots via root-specific aquaporins [19]. Although many abiotic transcriptome studies have been performed in *Arabidopsis thaliana* and rice, a significant differences in organ-specific transcript profiling has been observed [20]. Both of these types of plants clearly use distinct combinations of genes for abiotic stress responses and regulation.

In the present study, to gain insights into the key components that determine rice cold stress tolerance, we performed transcriptome analysis with a custom designed oligonucleotide array, a rice OneArray microarray platform (Phalanx Biotech Group Inc. [21]), using shoot and root tissues from TNG67 and TCN1 rice plants. Here, we discuss new findings from data analyses of DEGs, metabolic processes, changes in phytohormone-related gene expression, and important TFs.

## Materials and Methods

### Plant materials

Seeds of the rice cultivars TCN1 and TNG67 were obtained from the Taichung and Kaohsiung experimental research stations in Taiwan. Seeds were immersed in water for germination and then hydroponically cultivated in Kimura B solution at pH 5.0. Seedlings were grown in a phytotron at 30°C day/25°C night and 70~80% relative humidity. The plants were transferred to a growth chamber at 4°C for a cold treatment after growth to the three-leaf stage. After a 3- or 24-hr cold treatment or recovery to normal growth conditions for 24 hr, rice roots and shoots were harvested and immediately frozen in liquid nitrogen for RNA extraction.

### Measurement of electrolytes and Fv/Fm

For electrolyte leakage assay, shoots and roots of five rice seedlings were washed and immersed in deionized water and then kept in the dark for 24 hr at 25°C. The amount of electrolytes that leached into the solution was measured with a conductivity meter (Extech EC500 Waterproof ExStik pH/Conductivity Meter). The change in conductivity was calculated as the ratio of the percentages of electrolyte leakage before and after autoclaving. Conductivity after autoclaving is assumed to represent complete (100%) electrolyte leakage. The maximum quantum efficiency of PSII photochemistry (Fv/Fm) was recorded as an indicator of PSII capacity. A JUNIOR-PAM chlorophyll fluorometer (Heinz Walz) was used to determine Fv/Fm at a position located 2 cm from the leaf tip of the third leaf of each rice seedling with or without cold treatment at various time points.

### Microarray analysis

Transcriptome analysis was conducted using a 22-k Rice Whole Genome OneArray v1 oligo microarray platform (Phalanx Biotech Group, Inc., Taiwan), which was designed with high specificity and sensitivity for both *japonica* and *indica* rice. This array was constructed using MSU v6.1 database cDNA sequences, BGI *indica* (93–11) coding sequences and BGI *japonica* (Syngenta) coding sequences. Probes were designed and selected to cover 90% of the well-annotated genes found in both rice subspecies. The chip contains 22,003 probes that are randomly distributed across the array, each comprising a 60-mer oligonucleotide. In addition to 824 quality control probes, 21,179 probes covering 20,806 and 13,683 genes in *japonica* and *indica* were present on the chip, which has been well designed and validated [11]. After combining protein-coding and non-protein-coding loci with rice FLcDNA, the exact number of genes shared by both cultivars was determined to be 13119. The gene array list with detailed descriptions can be found at http://www.phalanx.com.tw/products/RiOA_Probe.php.

Total RNA extractions from shoots and roots were performed using TRIzol reagent (Invitrogen) according to manufacturer’s instructions. RNA quality was determined based on 260/280 ratio and a 260/230 ratio of > 2.0. One microgram of total RNA was reverse transcribed and amplified using an Amino Allyl aRNA Amplification Kit (Phalanx Biotech Group, Taiwan) and labeled with Cy5 dyes (Amersham Pharmacia, Piscataway, NJ, USA). Fluorescent targets were hybridized to the rice whole-genome OneArray with Phalanx hybridization buffer using a Phalanx Hybridization System. After 16 hr of hybridization at 50°C, non-specific binding targets were washed away via three different washing steps (wash I, 42°C for 5 min; Wash II, 42°C for 5 min and 25°C for 5 min; and Wash III, rinse 20 times). The arrays were scanned using a GenePix 4200A 01 Autoloader (635 nm, power of 100, and PMT of 520; and 532 nm, power of 10, and PMT of 460), and fluorescence intensity was quantified. The original dataset was deposited in the NCBI GEO database (accession no. GSE57895).

The signal intensity of each spot was loaded into a Rosetta Resolver System (Rosetta Biosoftware) for data analysis. The error model of this system allows for the removal of both systematic and random errors from data. We filtered out scanned spots with a flag value of < 0. Spots that passed the criteria were normalized by 50% media scaling normalization method. DEGs were considered those with changes in expression of greater than two-fold after normalization to the control and with significant results for the *t* test (*p* < 0.05) based on 6 replicates (3 biological repeats x 2 technical repeats) for each treatment compared with the control. These DEGs were verified by quantitative PCR (qPCR) and correlation analysis (R = 0.857; p < 0.05) (S1 Fig). The heuristic cluster method was used for clustering analysis.

### Quantitative RT-PCR

Integrated RNA was reverse transcribed into cDNA using SuperScript III Reverse Transcriptase (Invitrogen) for qPCR analysis. The synthesized cDNA was mixed with SYBR Green PCR Master Mix (Applied Biosystems), and real-time PCR was conducted with an Applied Biosystems ABI 7500 sequence detection system. The corresponding gene-specific primers used in gene expression analysis are listed in S1 Table.

### Hierarchical cluster analysis

Hierarchical cluster analysis of the DEGs in the shoots and roots was performed with Gene Cluster 3.0 (http://bonsai.hgc.jp/~mdehoon/software/cluster/) followed by Alok Saldanha’s Java TreeView v1.1.6r2 for graphical analysis of the results.

### MapMan analysis

The averaged signals for the cold treatment (3 biological replicates x 2 technical replicates) at different time points were expressed relative to those of the control samples, converted to a log_2_ scale and displayed using MapMan v3.5.1 (http://gabi.rzpd.de/projects/MapMan/) [22].

### Network analysis of co-expressed genes

Microarray network analysis was performed using Rice Co-expression Network (http://www.ricearray.org/coexpression/network.shtml). The network type selected was “Abiotic stress”, the correlation coefficient cutoff was set at 0.8, and the depth to search co-expressed genes was set at 2. After online analysis, an SIF file was created and downloaded. We used Cytoscape v3.1.1 (http://www.cytoscape.org/) to visualize and edit the SIF file and generate graphs.

## Results

### Physiological and phenotype analyses showed that TNG67 is a cold-tolerant rice variety

**Fig 1:**
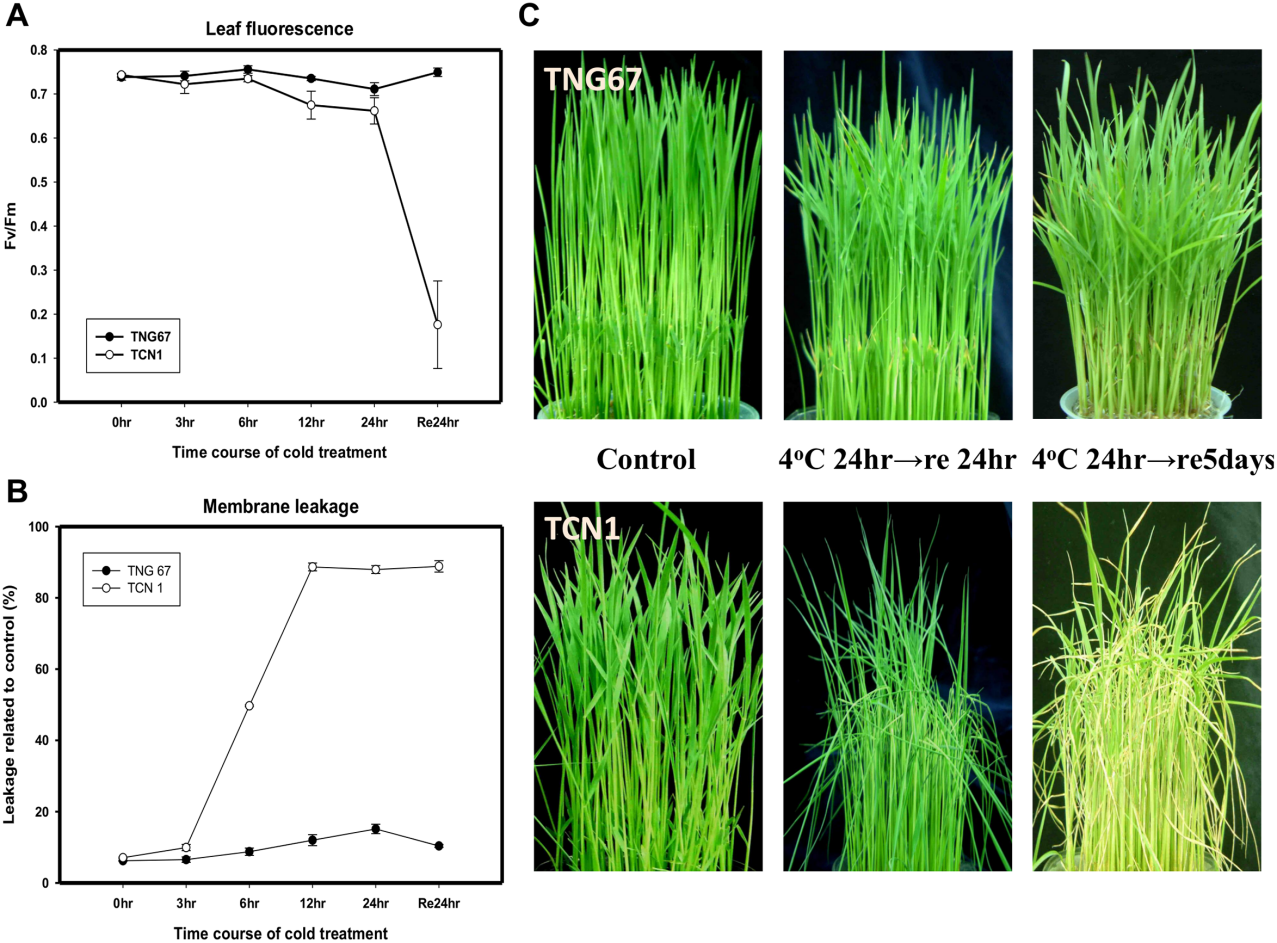
The injury severity test for the measurements of (A) Fv/Fm, (B) leakage conductivity and phenotype performance (C) under normal growth (0 hr) and different durations of cold exposure (4°C) followed by recovery at 30°C/25°C for 1 or 5 days.

TNG67 and TCN1 show contrasting cold tolerance at 4°C [4]. To assess the effect of cold on photosynthetic capacity, we measured Fv/Fm values in leaves of TNG67 and TCN1 rice plants. After recovery from the cold for 24 hr, the Fv/Fm values were low in the TCN1 rice leaves but were similar to those measured under normal growth conditions in the TNG67 leaves (Fig 1A). To dissect the differences in the dynamic cold response between TNG67 and TCN1, we measured electrical conductivity and Fv/Fm parameters in both types of rice seedlings after various durations of cold treatment. The membrane leakage of TCN1 rice seedlings immediately increased following cold treatment for 3 hr and plateaued at 12 hr. However, the electrical conductivity of TNG67 rice seedlings remained relatively unchanged (Fig 1B). In response to cold treatment, the TCN1 but not the TNG67 leaves were pale, wilted and chlorotic (Fig 1C). Therefore, the TCN1 rice was more sensitive to low temperatures during the early phase (3 hr) and lost viability after 5 days of recovery.

### Re-establishment of homeostasis during recovery is important, and shoots or roots may play a distinct role in regulating cold stress tolerance in rice

**Fig 2:**
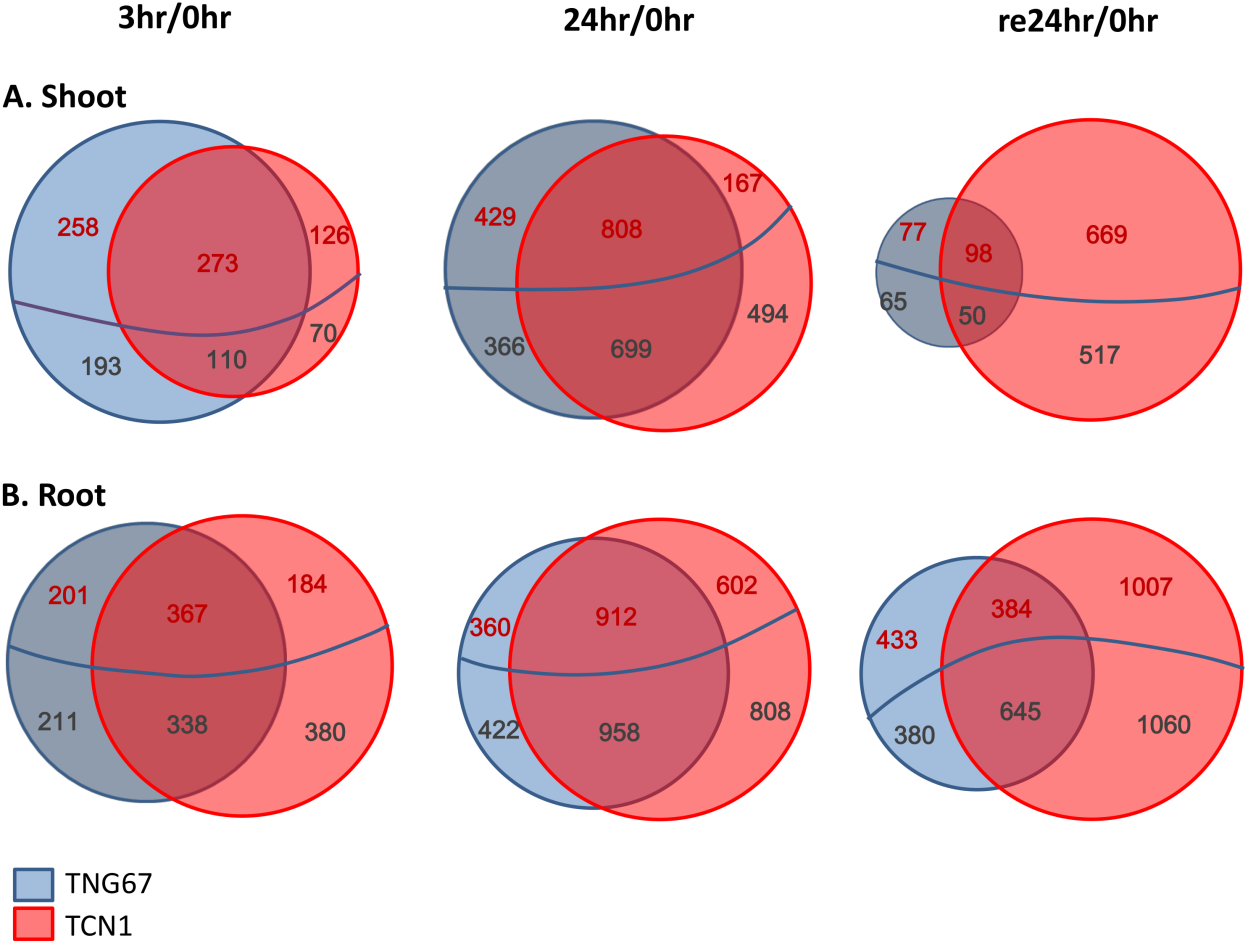
Venn diagram of DEGs in the shoots and roots of TNG67 and TCN1 rice seedlings exposed to cold (3 and 24 hr) and allowed to recover for 1 day (24 hr). The upregulated genes are shown above the blue line with the expression levels written in red, while the downregulated genes are shown below the blue line with the expression levels written in dark grey. DEGs with significant expression changes in the shoots (A) and roots (B) of TNG67 and TCN1 rice seedlings. Genes with a greater than two-fold change in expression compared with the control and a significant *t* test (*p* < 0.05) result were considered DEGs.

To compare the global gene expression changes in the rice cultivars under cold stress, we performed comparative transcriptome analysis with a customer-created oligonucleotide microarray and RNA from roots or shoots of TNG67 and TCN1 rice seedlings at early (3 hr; C3) and late (24 hr; C24) cold treatment and recovery time points (return to normal growth for 24 hr; Re24). DEG analysis was conducted with 3 individual biological and technical replicates, and the gene expression results revealed greater than two-fold changes in the expression of a number of genes after normalization to the control and significant *t* test results (*p* < 0.05) (S1 and S2 Tables). Comparisons of gene expression between the shoots of the two cultivars revealed a total of 834 DEGs in TNG67 and 579 DEGs in TCN1 at C3. Among them, 383 DEGs were shared by both cultivars. Following 24 hr of cold stress (C24), 2032 DEGs were identified in TNG67 in addition to 2168 DEGs in TCN1, and the two varieties shared 507 DEGs. After recovery, 1334 DEGs were present in TCN1, but only 290 DEGs were detected in TNG67, and only 148 DEGs were shared by both varieties (Fig 2A). In contrast, 1117 and 1269 DEGs were identified in the roots of TNG67 and TCN1, respectively, at C3 (705 DEGs in common), and 2652 and 3280 were detected at C24, respectively (1870 DEGs in common). After recovery, we discovered 1842 DEGs in TNG67 and 3096 in TCN1, 1029 of which were shared by both cultivars (Fig 2B). In response to cold treatment, the number of DEGs identified in roots was greater than those in shoots in both TNG67 and TCN1 (Fig 2B). This result indicated that root transcript levels were more easily regulated compared with those in shoots. Thus, roots may be more responsive to cold stress than shoots in both cultivars. Additionally, we found markedly increased numbers of DEGs in the shoots (by approximately five-fold) and roots (by approximately two-fold) of TCN1 during recovery. The expression patterns of the DEGs in the shoots and roots suggest that these 2 different tissues may play distinct roles in regulating rice cold stress tolerance.

**Fig 3:**
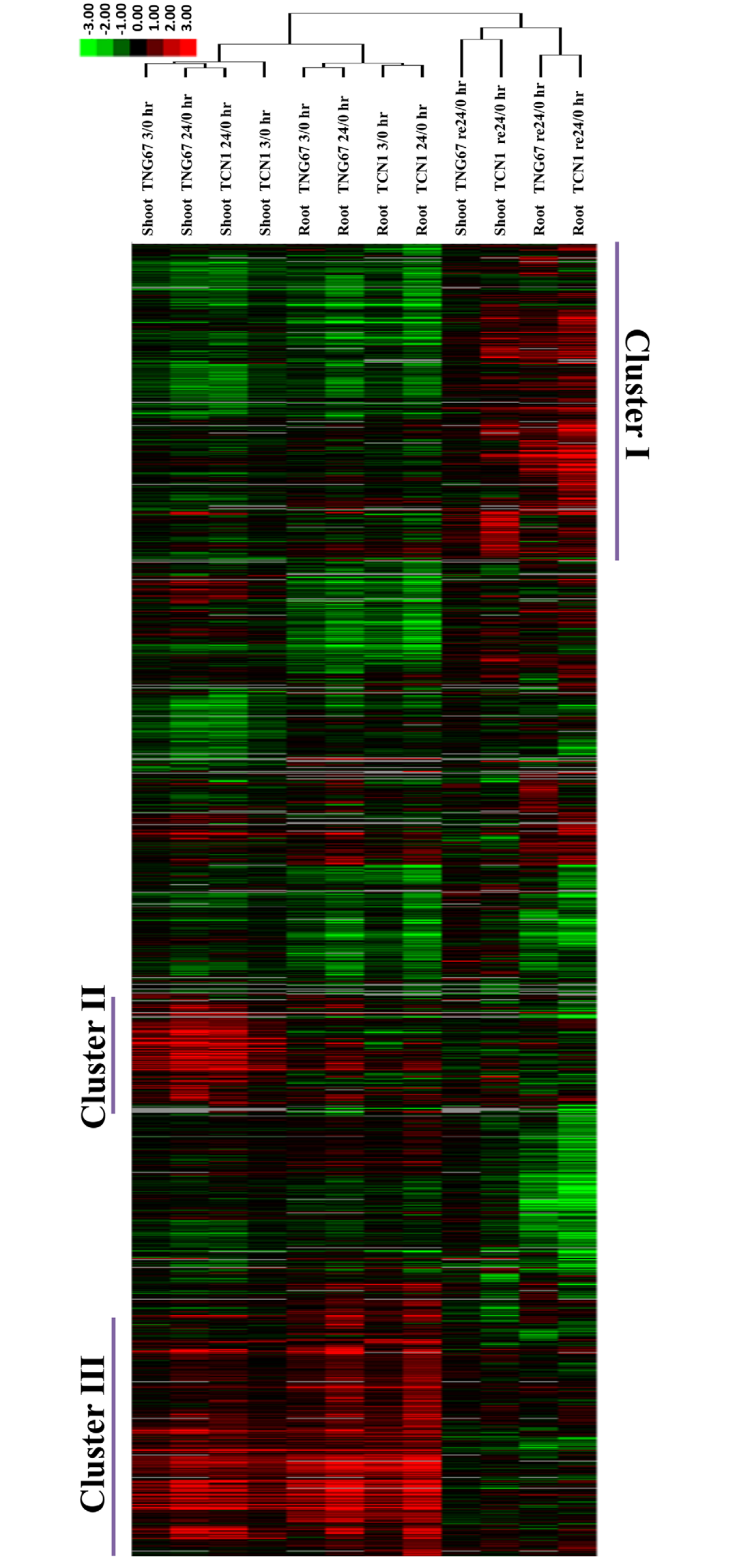
Hierarchical cluster analysis of DEGs in TNG67 and TCN1 shoots and roots after 3- and 24-hr cold stress treatments and a subsequent 24-hr recovery period.

Based on the similarity of the gene expression patterns between the roots and shoots of the two cultivars, we analyzed the microarray data using a hierarchical clustering method. After the 3- and 24-hr cold treatments and subsequent recovery of TNG67 and TCN1 shoots and roots, the DEGs could be separated into 3 distinct clusters (Fig 3). Cluster I contained those that were repressed under cold stress but induced after recovery. Cluster II comprised shoot but not root DEGs that were induced in response to cold stress. Cluster III included DEGs that were induced by cold in both shoots and roots. Cold treatment and recovery distinctly affected gene expression at various time points.

### Pathways that mediate protein metabolism, modification, translation, stress response, and cell death are important for cold stress tolerance in rice

To group DEGs with similar functions and identify functional categories with different DEGs in rice in response to cold stress, we performed gene ontology (GO) analysis of the microarray dataset. The numbers of DEGs in almost every GO Slim category of biological process were higher in TNG67 than in TCN1 in response to cold stress.

In shoots (Table 1), some of the GO Slim categories with upregulated genes were significantly enriched in TNG67 but not in TCN1 during the early stage (3 hr) of cold stress, including protein metabolic process (GO:0019538), protein modification process (GO:0006464), response to stress (GO:0006950), and cell death (GO:0016265). GO:0006091 (generation of precursor metabolites and energy) was an exception in TCN1, in which it was significantly enriched compared with TNG67. During the late stage of cold stress (C24), the numbers of upregulated genes in each GO Slim category were higher in TNG67 than in TCN1. However, the numbers of downregulated genes in each GO category were lower in TNG67 than in TCN1. After recovery, the DEGs in many GO Slim categories remained significantly enriched in TCN1. During recovery, only GO:0000160 [two-component signal transduction system (phosphorelay)] was significantly enriched among the upregulated genes in TNG67 and may be important for maintaining homeostasis during recovery (S4 Table). GO:0006979 (response to oxidative stress), GO:0042221 (response to chemical stimulus) and GO:0005975 (carbohydrate metabolic process) were significantly enriched among the downregulated genes in TCN1. Malfunctions in these processes during recovery may lead to death following cold stress.

**Table 1:** Gene ontology (GO) annotation of differentially expressed genes (DEGs) assigned to the biological process (BP) category after cold treatment and recovery in shoots. The bars show the numbers of genes in each GO Slim category. GO enrichment analysis was conducted using agriGO (http://bioinfo.cau.edu.cn/agriGO/), and GO terms with a p < 0.05 were considered to have significantly enriched expression in the cluster.

**Shoot**							
**TNG67**				**TCN1**			
**Up-regulated**							
**3/0 hr**							
**Term**	**Count**	**%**	**P-value**	**Term**	**Count**	**%**	**P-value**
cellular process	228	40.86%	4.10E-19	biosynthetic process	94	20.94%	2.40E-14
biosynthetic process	118	21.15%	6.40E-18	generation of precursor metabolites and energy	23	5.12%	8.00E-14
nucleobase, nucleoside, nucleotide and nucleic acid metabolic process	92	16.49%	3.40E-15	cellular process	175	38.98%	1.40E-13
metabolic process	235	42.11%	1.30E-13	photosynthesis	13	2.90%	7.60E-11
photosynthesis	8	1.43%	5.70E-05	nucleobase, nucleoside, nucleotide and nucleic acid metabolic process	69	15.37%	1.40E-10
protein modification process	49	8.78%	8.50E-05	metabolic process	182	40.53%	7.20E-10
generation of precursor metabolites and energy	11	1.97%	0.0012	cellular component organization	14	3.12%	8.10E-05
cellular component organization	13	2.33%	0.002	transport	28	6.24%	0.019
death	12	2.15%	0.013	carbohydrate metabolic process	18	4.01%	0.021
protein metabolic process	71	12.72%	0.016				
response to stress	18	3.23%	0.017				
**24/0 hr**							
**Term**	**Count**	**%**	**P-value**	**Term**	**Count**	**%**	**P-value**
cellular process	510	39.66%	1.60E-38	cellular process	409	39.56%	7.00E-31
metabolic process	557	43.31%	8.50E-33	biosynthetic process	211	20.41%	3.20E-29
biosynthetic process	249	19.36%	2.80E-31	metabolic process	453	43.81%	1.10E-27
nucleobase, nucleoside, nucleotide and nucleic acid metabolic process	199	15.47%	2.90E-28	nucleobase, nucleoside, nucleotide and nucleic acid metabolic process	166	16.05%	3.10E-25
protein modification process	127	9.88%	2.70E-13	generation of precursor metabolites and energy	29	2.80%	7.80E-11
protein metabolic process	192	14.93%	1.80E-08	Photosynthesis	15	1.45%	2.50E-08
generation of precursor metabolites and energy	27	2.10%	1.30E-07	protein modification process	91	8.80%	9.70E-08
transport	84	6.53%	2.40E-05	catabolic process	27	2.61%	9.80E-06
cellular component organization	25	1.94%	0.00033	protein metabolic process	143	13.83%	3.70E-05
catabolic process	27	2.10%	0.00033	cellular component organization	22	2.13%	0.00023
photosynthesis	10	0.78%	0.00093	carbohydrate metabolic process	41	3.97%	0.00098
carbohydrate metabolic process	48	3.73%	0.0013	Transport	61	5.90%	0.0029
lipid metabolic process	24	1.87%	0.0049	lipid metabolic process	18	1.74%	0.025
response to stress	34	2.64%	0.022				
signal transduction	17	1.32%	0.031				
**re24/0 hr**							
**Term**	**Count**	**%**	**P-value**	**Term**	**Count**	**%**	**P-value**
carbohydrate metabolic process	13	7.39%	0.00041	metabolic process	342	44.02%	1.8E-21
metabolic process	66	37.50%	0.00087	carbohydrate metabolic process	56	7.21%	8.8E-13
catabolic process	6	3.41%	0.0097	cellular process	217	27.93%	3.9E-06
biosynthetic process	25	14.20%	0.0087	biosynthetic process	102	13.13%	7.9E-06
nucleobase, nucleoside, nucleotide and nucleic acid metabolic process	19	10.80%	0.019	nucleobase, nucleoside, nucleotide and nucleic acid metabolic process	82	10.55%	8.3E-06
				transport	53	6.82%	0.00028
				protein metabolic process	106	13.64%	0.00053
				generation of precursor metabolites and energy	13	1.67%	0.0019
				protein modification process	54	6.95%	0.0057
				catabolic process	16	2.06%	0.0061
				lipid metabolic process	16	2.06%	0.0085
				response to stress	22	2.83%	0.032
**Down-regulated**							
**3/0 hr**							
**Term**	**Count**	**%**	**P-value**	**Term**	**Count**	**%**	**P-value**
metabolic process	130	43.05%	1.2E-08	protein modification process	23	11.79%	0.00016
cellular process	108	35.76%	2E-07	nucleobase, nucleoside, nucleotide and nucleic acid metabolic process	27	13.85%	0.00024
nucleobase, nucleoside, nucleotide and nucleic acid metabolic process	45	14.90%	4.5E-07	metabolic process	75	38.46%	0.00023
protein modification process	37	12.25%	9.1E-07	biosynthetic process	32	16.41%	0.00042
biosynthetic process	52	17.22%	2.6E-06	cellular process	57	29.23%	0.0061
protein metabolic process	50	16.56%	0.00045	protein metabolic process	30	15.38%	0.013
lipid metabolic process	8	2.65%	0.015				
response to stress	11	3.64%	0.026				
**24/0 hr**							
**Term**	**Count**	**%**	**P-value**	**Term**	**Count**	**%**	**P-value**
metabolic process	386	36.28%	1.80E-13	metabolic process	444	37.19%	1.10E-16
protein modification process	101	9.49%	5.50E-10	cellular process	362	30.32%	8.30E-13
lipid metabolic process	32	3.01%	1.70E-07	transport	104	8.71%	2.70E-12
transport	81	7.61%	1.60E-07	protein modification process	114	9.55%	3.10E-11
response to stress	42	3.95%	5.30E-06	reproduction	18	1.51%	2.60E-10
reproduction	11	1.03%	3.10E-05	nucleobase, nucleoside, nucleotide and nucleic acid metabolic process	123	10.30%	1.70E-07
carbohydrate metabolic process	46	4.32%	8.10E-05	protein metabolic process	167	13.99%	4.90E-06
protein metabolic process	144	13.53%	8.60E-05	response to stress	45	3.77%	7.70E-06
cellular process	269	25.28%	0.00013	pollination	11	0.92%	1.10E-05
nucleobase, nucleoside, nucleotide and nucleic acid metabolic process	90	8.46%	0.003	lipid metabolic process	29	2.43%	3.30E-05
pollination	7	0.66%	0.0033	biosynthetic process	140	11.73%	3.50E-05
				death	28	2.35%	4.80E-05
				carbohydrate metabolic process	45	3.77%	0.0015
				cell communication	11	0.92%	0.004
**re24/0 hr**							
**Term**	**Count**	**%**	**P-value**	**Term**	**Count**	**%**	**P-value**
metabolic process	53	41.73%	0.00039	metabolic process	231	40.53%	4.2E-12
cellular process	43	33.86%	0.0019	cellular process	192	33.68%	2.8E-10
nucleobase, nucleoside, nucleotide and nucleic acid metabolic process	18	14.17%	0.0019	carbohydrate metabolic process	36	6.32%	2.2E-07
protein modification process	14	11.02%	0.005	protein modification process	56	9.82%	1.4E-06
transport	12	9.45%	0.0077	response to stress	27	4.74%	0.000013
				nucleobase, nucleoside, nucleotide and nucleic acid metabolic process	60	10.53%	0.00013
				lipid metabolic process	16	2.81%	0.00043
				biosynthetic process	70	12.28%	0.00099
				transport	38	6.67%	0.0027
				pollination	5	0.88%	0.0039
				generation of precursor metabolites and energy	10	1.75%	0.0044
				reproduction	5	0.88%	0.0096
				protein metabolic process	73	12.81%	0.013
				death	12	2.11%	0.015

In roots (Table 2), GO:0019538 (protein metabolic process), GO:0006464 (protein modification process), and GO:0006412 (translation) were significantly enriched at both the DNA and protein levels among the upregulated genes in TNG67 but were not or were less enriched in TCN1 after 3 hr of cold stress. After removal from the cold for 24 hr, the DEGs were significantly enriched in the GO:0006457 (protein folding), GO:0008643 (carbohydrate transport) and GO:0006952 (defense response) categories in TNG67 but not in TCN1 (S5 Table).

**Table 2:** GO annotation of DEGs assigned to the biological process (BP) category after cold treatment and recovery in roots. The bars show the numbers of genes in each GO Slim category. GO enrichment analysis was conducted using agriGO (http://bioinfo.cau.edu.cn/agriGO/), and GO terms with a p < 0.05 were considered to have significantly enriched expression in the cluster.

**Root**							
**TNG67**				**TCN1**			
**Up-regulated**							
**3/0 hr**							
**Term**	**Count**	**%**	**P-value**	**Term**	**Count**	**%**	**P-value**
biosynthetic process	119	20.95%	8.5E-18	biosynthetic process	110	19.78%	4.4E-15
cellular process	222	39.08%	6.2E-17	nucleobase, nucleoside, nucleotide and nucleic acid metabolic process	90	16.19%	1.7E-14
metabolic process	245	43.13%	5E-15	cellular process	206	37.05%	5.6E-14
nucleobase, nucleoside, nucleotide and nucleic acid metabolic process	91	16.02%	2.1E-14	metabolic process	229	41.19%	1.5E-12
cellular component organization	16	2.82%	0.000082	transport	44	7.91%	0.000044
protein modification process	48	8.45%	0.00023	cellular component organization	15	2.70%	0.00021
protein metabolic process	83	14.61%	0.00033	photosynthesis	7	1.26%	0.00037
transport	41	7.22%	0.00046	generation of precursor metabolites and energy	10	1.80%	0.0037
catabolic process	13	2.29%	0.0057	carbohydrate metabolic process	21	3.78%	0.024
translation	17	2.99%	0.011	protein modification process	37	6.65%	0.033
carbohydrate metabolic process	22	3.87%	0.017	protein metabolic process	68	12.23%	0.034
				cellular homeostasis	5	0.90%	0.036
**24/0 hr**							
**Term**	**Count**	**%**	**P-value**	**Term**	**Count**	**%**	**P-value**
cellular process	537	40.44%	2.3E-42	cellular process	650	40.45%	2.9E-51
biosynthetic process	280	21.08%	1.1E-40	metabolic process	718	44.68%	1.2E-45
metabolic process	586	44.13%	3.5E-36	biosynthetic process	322	20.04%	7.3E-43
nucleobase, nucleoside, nucleotide and nucleic acid metabolic process	199	14.98%	1E-26	nucleobase, nucleoside, nucleotide and nucleic acid metabolic process	230	14.31%	2.9E-28
translation	68	5.12%	1.8E-16	translation	93	5.79%	9.7E-26
protein metabolic process	220	16.57%	4.2E-13	generation of precursor metabolites and energy	48	2.99%	2.6E-18
generation of precursor metabolites and energy	33	2.48%	7.9E-11	protein metabolic process	268	16.68%	5.4E-16
protein modification process	110	8.28%	8.4E-08	photosynthesis	25	1.56%	6.9E-14
photosynthesis	16	1.20%	1E-07	catabolic process	47	2.92%	1.5E-10
catabolic process	33	2.48%	2.9E-06	cellular component organization	38	2.36%	1.1E-07
cellular component organization	28	2.11%	0.000039	transport	101	6.29%	0.000017
transport	83	6.25%	0.00011	carbohydrate metabolic process	64	3.98%	0.000039
carbohydrate metabolic process	50	3.77%	0.00088	protein modification process	111	6.91%	0.00014
response to stress	37	2.79%	0.0087	response to stress	46	2.86%	0.0023
				response to endogenous stimulus	5	0.31%	0.014
**re24/0 hr**							
**Term**	**Count**	**%**	**P-value**	**Term**	**Count**	**%**	**P-value**
metabolic process	378	45.38%	2.00E-25	metabolic process	632	44.95%	7.70E-41
cellular process	276	33.13%	1.90E-13	protein modification process	166	11.81%	6.20E-24
protein modification process	90	10.80%	1.20E-11	cellular process	466	33.14%	8.40E-22
protein metabolic process	133	15.97%	1.10E-07	carbohydrate metabolic process	83	5.90%	9.60E-14
carbohydrate metabolic process	46	5.52%	2.00E-07	protein metabolic process	226	16.07%	2.70E-12
biosynthetic process	110	13.21%	2.80E-06	nucleobase, nucleoside, nucleotide and nucleic acid metabolic process	152	10.81%	3.30E-10
nucleobase, nucleoside, nucleotide and nucleic acid metabolic process	83	9.96%	4.70E-05	biosynthetic process	186	13.23%	1.10E-09
death	22	2.64%	5.90E-05	lipid metabolic process	39	2.77%	5.80E-08
response to stress	32	3.84%	0.00011	secondary metabolic process	9	0.64%	6.50E-06
reproduction	8	0.96%	0.00063	reproduction	13	0.92%	1.90E-05
lipid metabolic process	19	2.28%	0.0015	catabolic process	31	2.20%	5.20E-05
photosynthesis	7	0.84%	0.0036	transport	87	6.19%	0.0001
pollination	6	0.72%	0.0042	response to stress	43	3.06%	0.00095
transport	44	5.28%	0.047	cell communication	13	0.92%	0.0017
				death	26	1.85%	0.0026
**Down-regulated**							
**3/0 hr**							
**Term**	**Count**	**%**	**P-value**	**Term**	**Count**	**%**	**P-value**
cellular process	205	37.27%	1.60E-18	nucleobase, nucleoside, nucleotide and nucleic acid metabolic process	119	16.41%	4.20E-19
death	35	6.36%	1.30E-16	cellular process	274	37.79%	5.40E-19
cell death	35	6.36%	1.30E-16	metabolic process	307	42.34%	1.40E-17
protein modification process	70	12.73%	1.50E-14	protein modification process	95	13.10%	9.80E-17
response to stress	39	7.09%	2.80E-12	biosynthetic process	134	18.48%	5.30E-16
metabolic process	206	37.45%	4.90E-12	reproduction	13	1.79%	1.3E-08
nucleobase, nucleoside, nucleotide and nucleic acid metabolic process	76	13.82%	9.80E-12	response to stress	38	5.24%	2.2E-08
biosynthetic process	84	15.27%	2.10E-09	death	26	3.59%	5.5E-08
protein metabolic process	89	16.18%	5.10E-07	cell death	26	3.59%	5.5E-08
reproduction	7	1.27%	0.0006	protein metabolic process	120	16.55%	8.8E-08
pollination	5	0.91%	0.0062	pollination	10	1.38%	9E-07
pollen-pistil interaction	5	0.91%	0.0062	pollen-pistil interaction	10	1.38%	9E-07
transport	31	5.64%	0.0082	cell communication	11	1.52%	0.00007
cell communication	6	1.09%	0.013	carbohydrate metabolic process	27	3.72%	0.014
signal transduction	8	1.45%	0.019	transport	39	5.38%	0.048
**24/0 hr**							
**Term**	**Count**	**%**	**P-value**	**Term**	**Count**	**%**	**P-value**
protein modification process	162	11.69%	5.6E-23	metabolic process	708	39.71%	2.7E-32
cellular process	454	32.76%	1.7E-20	protein modification process	214	12.00%	1.5E-31
metabolic process	520	37.52%	6.2E-20	cellular process	591	33.15%	2.2E-27
response to stress	77	5.56%	5.8E-17	response to stress	100	5.61%	4.8E-22
death	54	3.90%	1.1E-16	protein metabolic process	281	15.76%	4.4E-14
cell death	54	3.90%	1.1E-16	nucleobase, nucleoside, nucleotide and nucleic acid metabolic process	198	11.10%	7E-14
protein metabolic process	223	16.09%	3.5E-12	reproduction	24	1.35%	1.9E-12
nucleobase, nucleoside, nucleotide and nucleic acid metabolic process	152	10.97%	1.3E-10	biosynthetic process	235	13.18%	9.4E-12
biosynthetic process	179	12.91%	1.1E-08	death	51	2.86%	6.1E-11
reproduction	16	1.15%	1E-07	cell death	51	2.86%	6.1E-11
transport	94	6.78%	2.00E-06	pollination	17	0.95%	2.2E-08
carbohydrate metabolic process	61	4.40%	3.6E-06	pollen-pistil interaction	17	0.95%	2.2E-08
pollination	12	0.87%	8.3E-06	carbohydrate metabolic process	78	4.37%	2E-07
pollen-pistil interaction	12	0.87%	8.3E-06	lipid metabolic process	44	2.47%	2E-07
cell communication	15	1.08%	0.00015	cell communication	20	1.12%	7.2E-06
signal transduction	20	1.44%	0.0089	transport	102	5.72%	0.00041
lipid metabolic process	23	1.66%	0.02	signal transduction	24	1.35%	0.0099
**re24/0 hr**							
**Term**	**Count**	**%**	**P-value**	**Term**	**Count**	**%**	**P-value**
metabolic process	389	37.62%	2.2E-15	metabolic process	713	41.50%	3.90E-37
response to stress	59	5.71%	9.9E-14	cellular process	592	34.46%	6.80E-31
carbohydrate metabolic process	60	5.80%	5E-10	biosynthetic process	276	16.07%	1.70E-23
lipid metabolic process	36	3.48%	7.3E-10	nucleobase, nucleoside, nucleotide and nucleic acid metabolic process	218	12.69%	5.50E-21
transport	87	8.41%	7.9E-10	response to stress	93	5.41%	1.40E-19
biosynthetic process	144	13.93%	4.8E-09	carbohydrate metabolic process	96	5.59%	2.40E-14
cellular process	297	28.72%	8.3E-09	lipid metabolic process	54	3.14%	1.60E-12
nucleobase, nucleoside, nucleotide and nucleic acid metabolic process	108	10.44%	5.3E-07	protein modification process	141	8.21%	2.20E-09
protein modification process	74	7.16%	0.00068	transport	118	6.87%	5.40E-08
reproduction	7	0.68%	0.009	reproduction	17	0.99%	3.50E-07
catabolic process	18	1.74%	0.018	photosynthesis	17	0.99%	6.20E-07
signal transduction	15	1.45%	0.021	protein metabolic process	217	12.63%	8.00E-05
				DNA metabolic process	31	1.80%	0.00029
				pollination	9	0.52%	0.004
				generation of precursor metabolites and energy	20	1.16%	0.0085
				catabolic process	25	1.46%	0.042
				death	25	1.46%	0.044

### Expression of genes involved in TCA cycle, glycolysis, proteasome, phenylpropanoid and lignin biosynthesis pathways was significantly induced in TNG67 rice in response to cold stress

**Fig 4:**
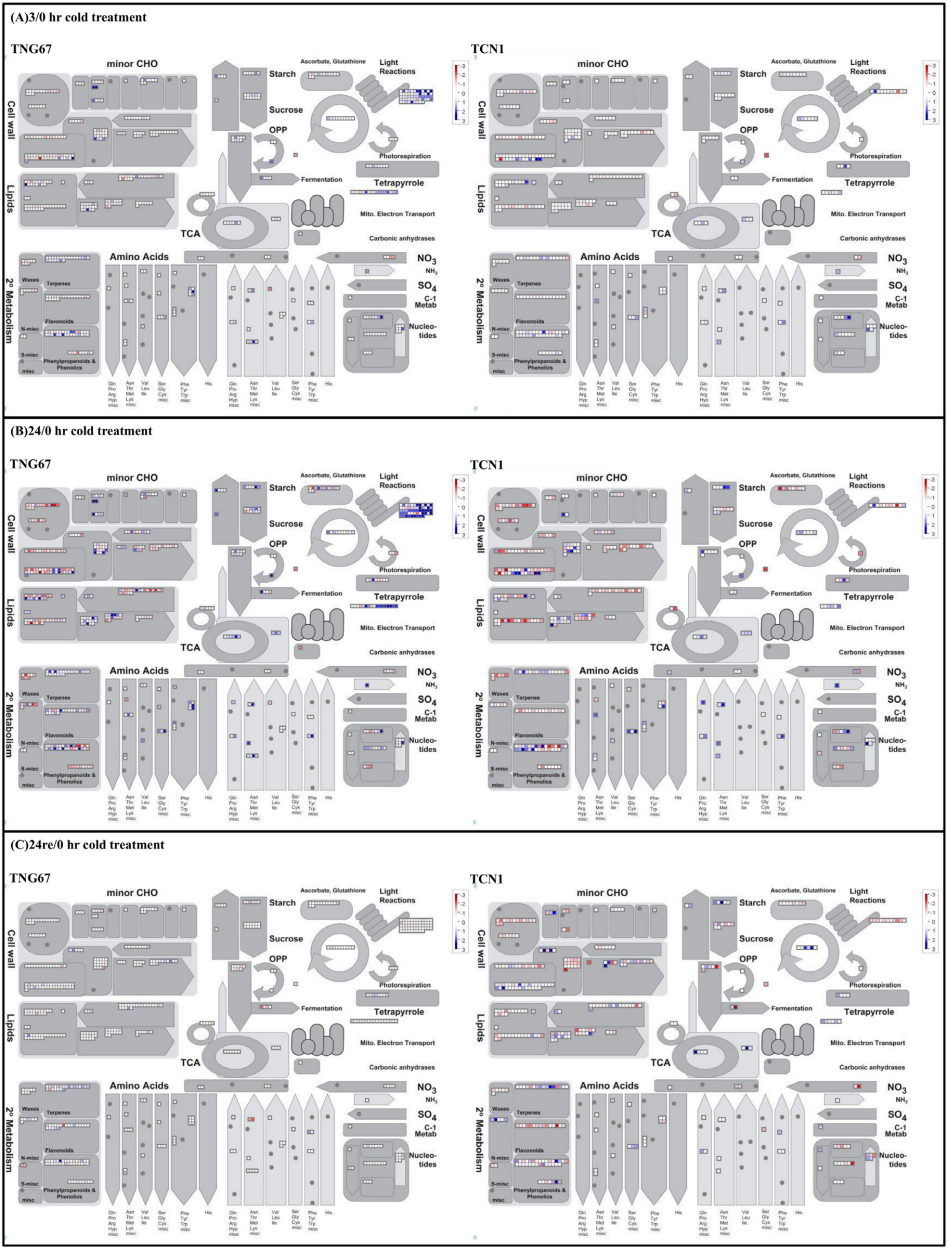
MapMan overview of changes in the expression of genes involved in metabolism in the shoots of rice exposed to cold for (A) 3 hr and (B) 24 hr and (C) following recovery for 24 hr after cold treatment. Genes involved in different metabolic processes are shown in the main panel in dark grey, while putatively associated genes are shown in light grey. Blue indicates that gene expression was induced and red indicates that gene expression was repressed compared with the control.

To further investigate alterations in metabolic pathways that occur during cold stress and recovery, we performed MapMan analysis to visualize the DEG data from microarray analysis. MapMan has been applied with Rice Net tools for systematic analysis of the early heat stress transcriptome in rice seedlings [23]. For shoots, the metabolic overview map (Fig 4) revealed a greater enhancement of gene expression associated with light reactions and mitochondrial electron transport (S6 Table) in TCN1 than in TNG67. In contrast, the expression of genes related to the TCA cycle and glycolysis pathways was elevated in TNG67 compared with TCN1. The detailed genes ID/description identified aforementioned are provided in EXCEL files as supplementary S6 Table. The proteasome map (S2 Fig) revealed the significantly increased expression of genes related to E2, E3 ligase, and the 20S proteasome in TNG67 compared with TCN1 following 3 hr of cold treatment. In particular, E2 ligase gene expression was predominant in TNG67 following 24 hr of treatment (S6 Table).

**Fig 5:**
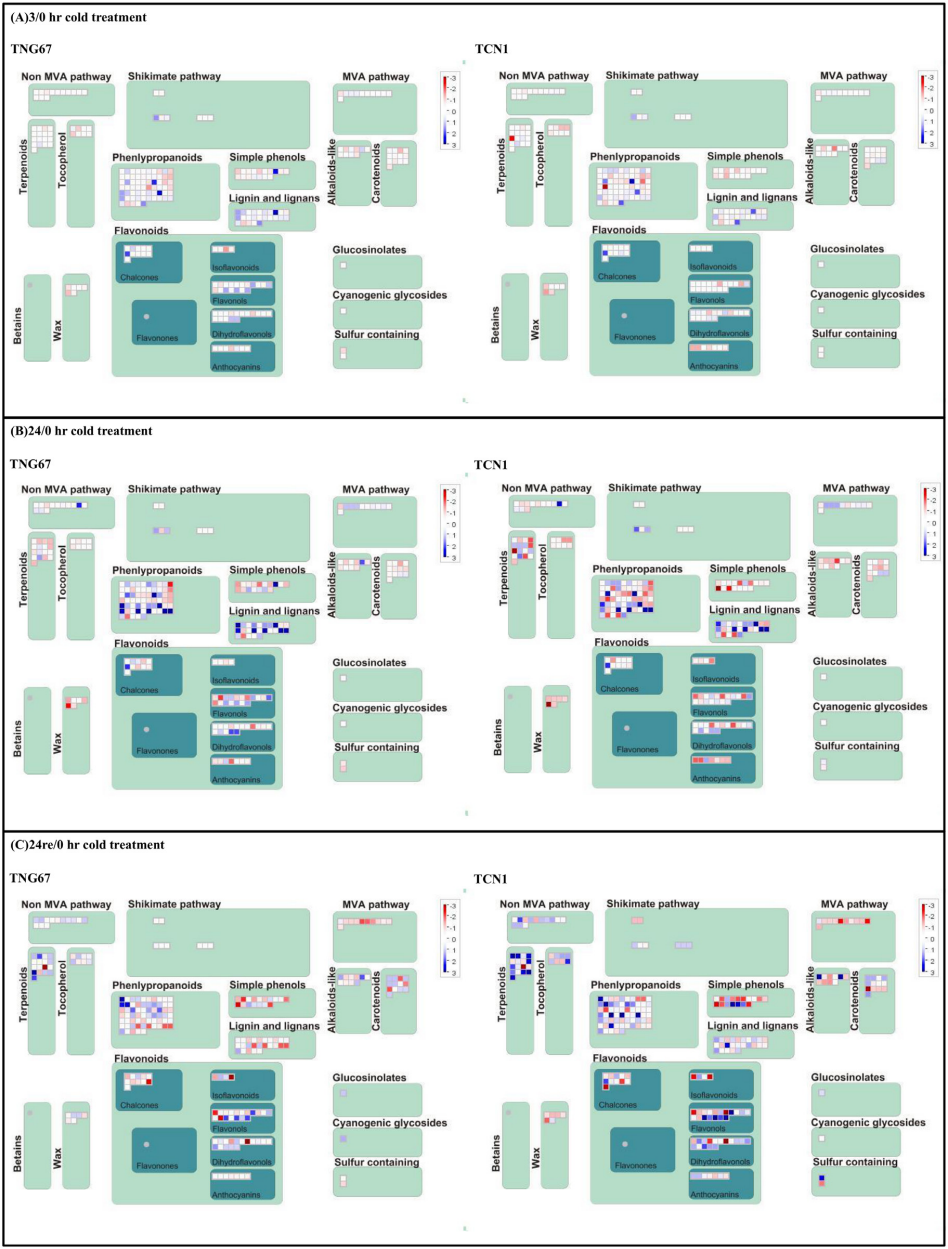
MapMan overview of changes in the expression of genes involved in the secondary metabolism of rice roots in response to cold treatment for (A) 3 hr and (B) 24 hr and (C) following recovery for 24 hr after cold treatment. Genes involved in different metabolic processes are shown in the main panel in dark green, while putatively associated genes are shown in light green. Blue indicates that gene expression was induced and red indicates that gene expression was repressed compared with the control.

In roots, the secondary metabolism map generated by MapMan analysis showed a tendency toward the increased activities of the phenylpropanoid and lignin biosynthesis (S6 Table) pathways in TNG67. Expression of the genes in these two pathways was greater in TNG67 than in TCN1 after 3 hr of cold stress. Additional genes were upregulated after 24 compared with those upregulated after 3 hr of cold stress in both cultivars; however, more genes were repressed in TCN1. In contrast, more genes were repressed in TNG67 after recovery compared with those repressed after 24 hr of cold stress (Fig 5).

### ABA, polyamines, auxin and jasmonic acid (JA)-related genes were preferentially expressed in TNG67 shoots and roots and were closely associated with cold stress tolerance

Plant hormone cross-talk occurs under various abiotic stresses and substantially regulates plant growth and development in response to stress [3, 24]. However, fluctuations in the levels of stress-responsive plant hormones occur during different stages, including biosynthesis, degradation, transportation, storage, perception and signal transduction. To obtain a better understanding of the roles of different plant hormones in rice in response to cold stress, we examined the expression profiles of various plant hormone-related genes in root and/or shoot tissues of rice cultivars using our microarray dataset.

### Expression profiles of ABA-related genes

**Fig 6:**
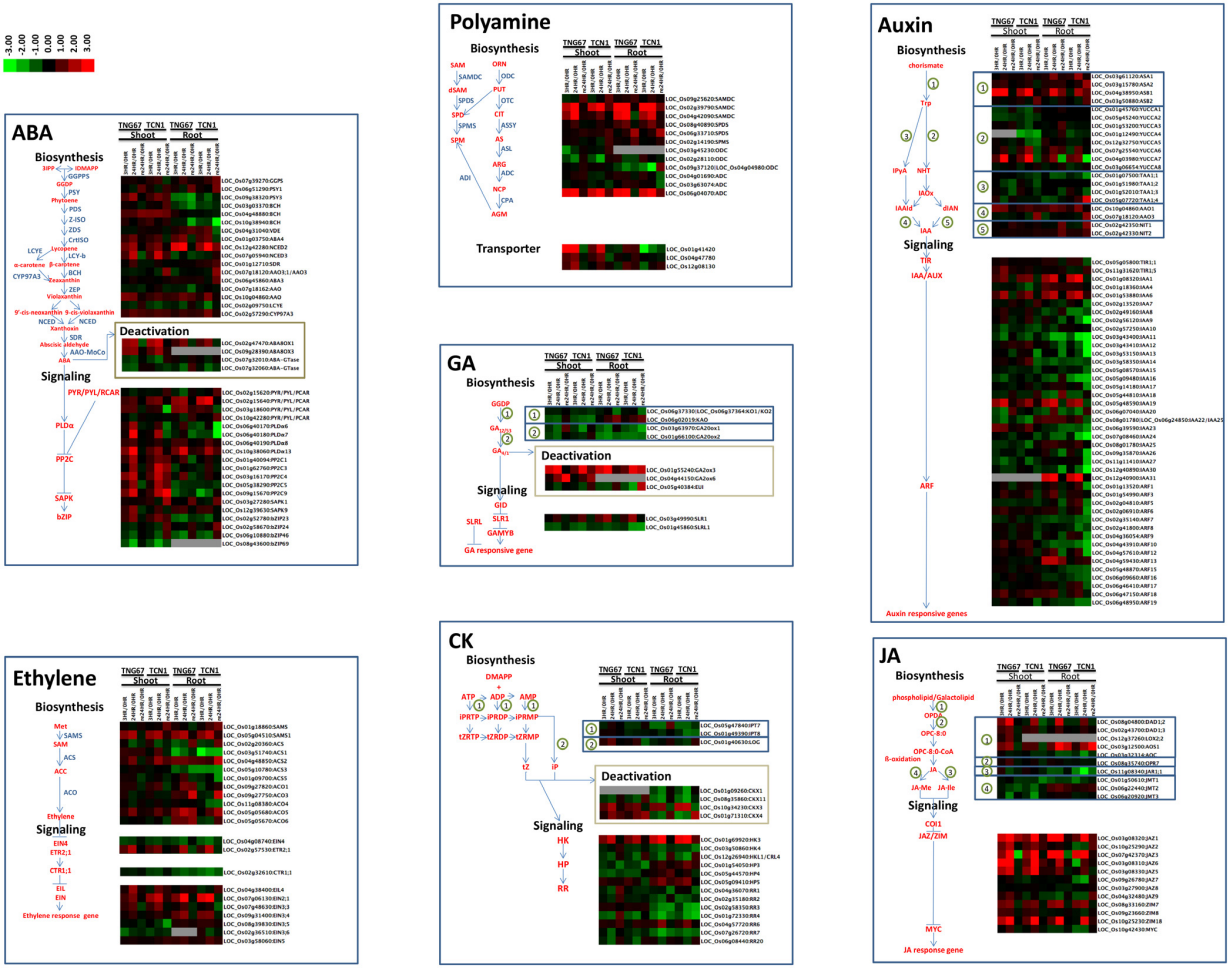
Representative gene expression profiles of hormone biosynthesis and deactivation signaling pathways in TNG67 and TCN1 shoots and roots. Only DEGs with significant changes in gene expression are shown in the heat map. Each color spot reflects the expression of a corresponding gene. Red indicates high levels of gene expression, green represents low levels, and gray indicates that no signal was detected on the microarray chip for the assessed gene probe.

ABA is a major stress hormone involved in plant responses to abiotic stresses. Previously, the ABA concentration has been found to rapidly increase in the shoots and roots of TNG67 but not in those of TCN1 in response to cold stress [5]. However, the molecular mechanism underlying the involvement of ABA in the cold stress response has not been well characterized. Based on our microarray analysis (Fig 6), the expression of LOC_Os09g38320 (PSY3) and LOC_Os01g12710 (SDR) was induced by cold temperatures to a greater extent in the shoots of TNG67 compared with the levels observed in those of TCN1. *PSY3* expression induces ABA biosynthesis under abiotic stresses [25]. These genes may contribute to the rapid biosynthesis and response of ABA in TNG67 shoots under cold stress. In TNG67, LOC_Os01g03750 (ABA4) and LOC_Os12g42280 (NCED2) transcripts were highly induced at low temperatures in both shoots and roots compared with TCN1. In addition, the expression levels of LOC_Os06g40170 (PLDα6), LOC_Os06g40180 (PLDα7), LOC_Os06g40190 (PLDα8), LOC_Os10g38060 (PLDα13), and LOC_Os12g39630 (SAPK9), which are involved in the ABA signal transduction pathway, were higher in the shoots of TNG67 than in those of TCN1. Two ABA-glucosyltransferase (ABA-GTase) genes, LOC_Os07g32010 and LOC_Os07g32060, which convert the storage form of ABA into its free form, were downregulated in TNG67 shoots. Furthermore, the expression levels of LOC_Os02g47470 (ABA8OX1) and LOC_Os09g28390 (ABA8OX3), which encode different isoforms of ABA 8'-hydroxylase genes responsible for the deactivation of ABA, were higher in shoots compared with roots. Therefore, the transcriptional regulation of ABA-related gene expression is one factor that controls cold stress tolerance in TNG67.

### Expression profiles of ET-related genes

Lee et al. [4] have shown that ACC and ET levels are decreased in TNG67 and TCN1 in response to cold treatment, and the ET level is increased after recovery in TNG67, but not in TCN1. Our microarray data (Fig 6) showed that most ET biosynthesis and signaling genes were upregulated in response to cold. ET biosynthesis-related genes, including LOC_Os05g04510 (SAMS1), LOC_Os04g48850 (ACS2) and LOC_Os05g05680 (ACO5) and the signaling genes LOC_Os02g57530 (ETR2; 1) and LOC_Os07g06130 (EIN2; 1), were significantly upregulated in both cultivars and tissues. Some ET biosynthesis genes, such as LOC_Os09g27820 (ACO1), LOC_Os05g05680 (ACO5) and LOC_Os05g05670 (ACO6), were upregulated in TNG67 roots at low temperatures and displayed prolonged expression during recovery. Therefore, the cold stress–mediated ET response may not be regulated at the transcriptional level in rice.

### Expression profiles of polyamine-related genes

Polyamines are abiotic stress modulators that interact with ABA and ET to affect plant abiotic stress tolerance [26]. Put is specifically involved in freezing tolerance and cold acclimation in *Arabidopsis* by regulating ABA levels in response to cold stress [27]. Thus, we assessed the expression profiles of polyamine-related genes. In response to cold stress, the levels of Put and ADC activity were increased in both the shoots and roots of TNG67. Furthermore, spermidine/spermine and SAMDC levels were increased in the shoots of TNG67 and were increased to a lesser extent in the shoots of TCN1 [6]. The expression of ADC genes, including LOC_Os06g04070 (ADC) and LOC_Os08g33620|LOC_Os06g04070 (ADC), was highly induced in TNG67 compared with TCN1 in both shoots and roots. The expression pattern of SAMDC genes, including LOC_Os02g39790 and LOC_Os04g42090, was similar to that of ADC genes. In addition, the expression of polyamine transporter genes in shoots, including LOC_Os01g41420, LOC_Os04g47780 and LOC_Os12g08130, was higher in TNG67 than in TCN1, particularly after 24 hr of cold stress (Fig 6). Therefore, the ADC-mediated pathway of Put biosynthesis appears to be an important mechanism of cold tolerance in TNG67.

### Expression profiles of gibberellin-related genes

Reductions in the endogenous gibberellic acid (GA) level [28] and DELLA activity [29] enhance the survival of *Arabidopsis* during salt stress. However, whether GA is linked to cold stress tolerance in rice is unknown. We examined the expression of GA-related genes and found the suppressed expression of GA biosynthesis-related genes, including LOC_Os06g02019 (KAO), LOC_Os03g63970 (GA20ox1), and LOC_Os01g66100 (GA20ox2), under cold conditions; in particular, LOC_Os01g66100 (GA20ox2) expression was more markedly downregulated in TNG67 than in TCN1 in both shoots and roots. In contrast, the decreased expression of genes in shoots, including LOC_Os01g55240 (GA2ox3) and LOC_Os04g44150 (GA2ox6), was upregulated in TNG67 compared with TCN1 (Fig 6). Thus, the reduction in GA concentration via transcriptional gene regulation may enhance cold stress tolerance in TNG67.

### Expression profiles of cytokinin (CK)-related genes

Recent data have suggested that the cross-talk between ABA and CK, and particularly the ABA:CK ratio in xylem sap, are important for stress signaling [30]. In addition, the CK two-component system is involved in the stress response to low temperatures in *Arabidopsis* [31]. Thus, we evaluated the CK-related gene expression profile in our array dataset. In the CK biosynthetic pathway, we identified only one gene, LOC_Os01g40630 (CK riboside 5'-monophosphate phosphoribohydrolase, LOG), that was induced in TNG67 shoots after 24 hr of cold stress. With regard to CK degradation, we discovered 2 catabolic enzymes, the CKX (CK dehydrogenase)-related genes LOC_Os10g34230 (CKX3) and LOC_Os01g71310 (CKX4), that were expressed at higher levels in the shoots of TNG67 compared with those of TCN1 in response to cold stress. However, LOC_Os10g34230 (CKX3) was preferentially induced in TCN1 roots. The expression of the majority of the genes associated with the CK signal transduction pathway was preferentially repressed in TNG67 compared with TCN1, including LOC_Os04g36070 (RR1) and LOC_Os07g26720 (RR7) in shoots and LOC_Os03g50860 (HK4), LOC_Os02g58350 (RR3), LOC_Os01g72330 (RR4) and LOC_Os07g26720 (RR7) in roots (Fig 6). CK may participate in antagonistic cross-talk with ABA to negatively regulate cold stress tolerance in rice. During the recovery stage, the CK synthesis gene LOC_Os05g47840 (IPT7) was upregulated in the roots of TNG67 but not in those of TCN1; however, this gene was downregulated in response to cold stress in both cultivars. In shoots, the gene expression profile of CK signal transduction, including the OsRR type-A genes LOC_Os04g36070 (RR1), LOC_Os02g35180 (RR2), LOC_Os01g72330 (RR4), LOC_Os04g57720 (RR6) and LOC_Os11g04720 (RR10), showed a similar pattern.

### Expression profiles of auxin-related genes

Auxin is tightly linked to cold stress-induced changes in plant growth and development [32]. Endogenous levels of indoleacetic acid (IAA) and JA are differentially modulated in rice under abiotic stress [33]. We analyzed the expression of genes related to auxin biosynthesis and found that LOC_Os04g38950 (ASB1) and LOC_Os04g03980 (YUCCA7) were induced under cold treatment in both tissues of the rice cultivars. LOC_Os07g25540 (YUCCA6) was induced in both tissues of TCN1 but only in the roots of TNG67. In contrast, LOC_Os10g04860 (AAO1) was upregulated in TNG67 shoots and roots but was increased only in TCN1 roots. Increased auxin expression under low temperatures may help to ameliorate cold injury in rice. Furthermore, the expression of the auxin signaling-related genes LOC_Os01g08320 (IAA1), LOC_Os01g53880 (IAA6), LOC_Os05g48590 (IAA19), and LOC_Os06g47150 (ARF18) was induced in both cultivars and tissues. The expression of most of these genes was higher in TNG67 than in TCN1. Other genes, such as LOC_Os05g05800 (TIR1; 1), were induced in TNG67 shoots and roots [LOC_Os04g59430 (ARF13) and LOC_Os01g18360 (IAA4)] (Fig 6). Thus, auxin expression may be higher in the shoots and roots of TNG67 than in those of TCN1 under cold stress. Interestingly, GUS staining was increased in the roots of DR5-GUS transgenic rice seedlings treated with cold stress (S3 Fig).

### Expression profiles of jasmonate-related genes

JA enhances the freezing tolerance of *Arabidopsis* by positively regulating the ICE-CBF/DREB1 pathway [34]. We dissected the JA-related gene expression profile in response to cold stress in rice. The levels of JA biosynthesis genes, including LOC_Os08g04800 (DAD1; 2), LOC_Os12g37260 (LOX2; 2) and LOC_Os08g35740 (OPR7), were higher in TNG67 than in TCN1 in shoots, and that of LOC_Os03g12500 (AOS1) was increased in roots. LOC_Os12g37260 (LOX2; 2) was the only gene that was specifically expressed in TNG67 shoots. These findings may indicate a pivotal role of JA synthesis and the contribution of increased JA content in triggering cross-talk between JA and cold stress. Our results are consistent with a previous report [34] demonstrating higher JA signaling-related expression [LOC_Os03g08320 (JAZ1), LOC_Os10g25290 (JAZ2), LOC_Os07g42370 (JAZ3), LOC_Os03g08310 (JAZ6), LOC_Os04g32480 (JAZ9), and LOC_Os10g25230 (ZIM18)] in TNG67 than in TCN1 (Fig 6). The addition of 50 μM IBU (JA biosynthesis inhibitor) to hydroponically cultured TNG67 and TCN1 rice seedlings in response to cold stress for 24 hr induced the hypersensitivity to cold stress of both cultivars (S4 Fig).

### NAC, WRKY and ERF/AP2 TFs are good candidates for involvement in cold stress tolerance

**Fig 7:**
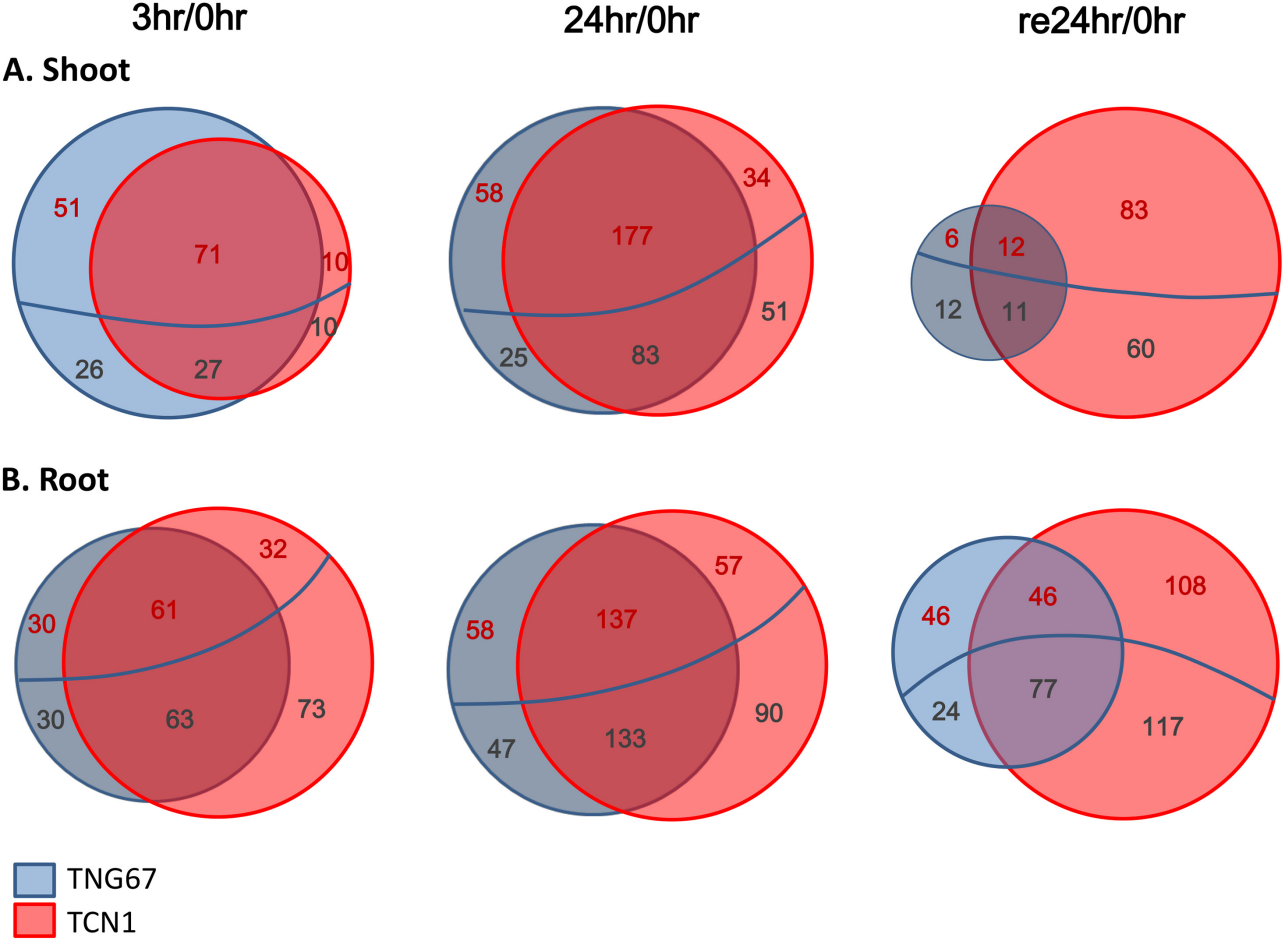
Venn diagram of upregulated (upper area above the blue line, expression levels written in red) and downregulated transcription factors (TFs) (area below the blue line, expression levels written in dark grey) in (A) the shoots and (B) roots of TNG67 and TCN1 at different time points of cold stress. Genes with a greater than two-fold change in expression compared with the control and a significant *t* test (*p* < 0.05) result were considered DEGs.

**Fig 8:**
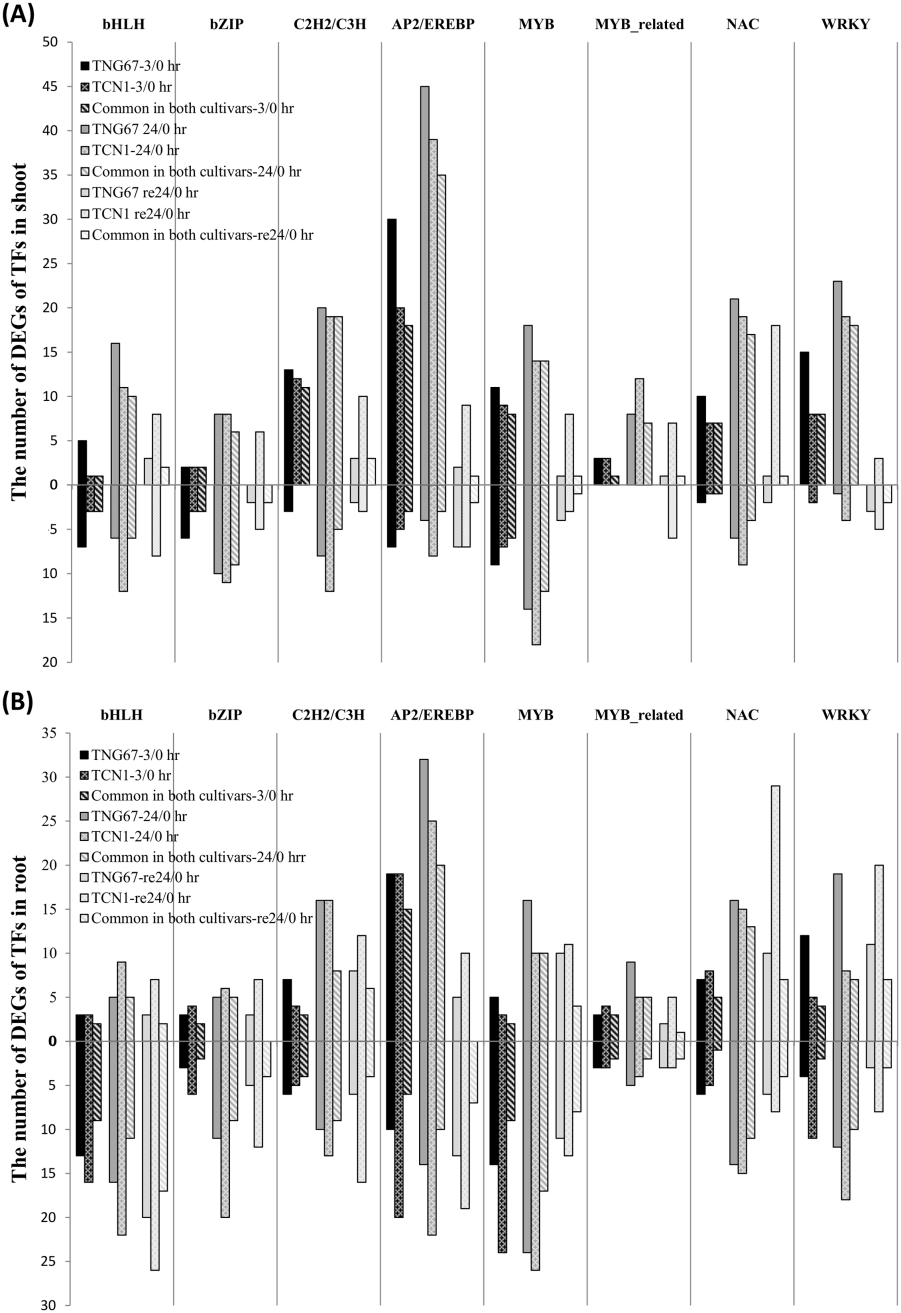
Numbers of major TF-encoding DEGs identified in TNG67 and TCN1 in response to cold stress and recovery in shoots (A) and roots (B). The Arabic numerals above the vertical axis of the graph are the sums of the different up-regulated TFs, while the Arabic numerals below the vertical axis of the graph are the sums of the different down-regulated TFs.

The expression of plant TFs is typically quickly altered in response to environmental stimuli, leading to the establishment of a pleiotropic phenotype to enhance stress tolerance. To elucidate the transcriptional regulation of cold stress–responsive genes, we identified DEGs encoding TFs under cold stress. For Venn diagram analysis, we determined the gene expression patterns of DEGs encoding TFs according to the Database of Rice Transcription Factors (http://drtf.cbi.pku.edu.cn/) (Fig 7). Following 3 hr of cold treatment, TNG67 and TCN1 shared 71 up- and 27 downregulated TF-encoding DEGs in shoots and 61 up- and 63 downregulated TF-encoding DEGs in roots. While 51 upregulated and 26 downregulated TFs were uniquely expressed in the shoots of TNG67, only 10 each of upregulated and downregulated TFs were uniquely expressed in those of TCN1. However, after 24 hr of cold stress, 30 upregulated and 30 downregulated TFs were uniquely expressed in TNG67 roots, and 32 upregulated and 73 downregulated TFs were uniquely expressed in TCN1 roots. Sixty-one up- and 63 down-regulated DEGs were shared by both cultivars. After 24 hr of cold treatment, greater numbers of TF-encoding DEGs (260 and 270 in the corresponding shoots and roots) were observed in these 2 cultivars. TNG67 and TCN1 shared fewer TF-encoding DEGs during the recovery stage. Twenty-three and 123 common DEGs were shared in the shoots and roots, respectively, of TNG67 and TCN1 (Fig 7). The detailed genes list of aforementioned TFs can be found in supplementary tables (S7 and S8 Tables). Nevertheless, TCN1 possessed more TF-encoding DEGs during the recovery stage. TNG67 only showed 6 up- and 12 downregulated TFs in shoots compared with TCN1. TNG67 roots displayed the specific expression of 46 up- and 24 downregulated TFs. Only one NF-YB TF (LOC_Os03g29970) was upregulated in both the shoots and roots of TNG67. In addition, we stringently selected TF-encoding DEGs that were specifically or preferentially expressed in TNG67 (induced or repressed by greater than two-fold compared with TCN1) in response to the cold treatment and subsequent recovery (Fig 7). We identified 45 and 30 TFs in shoots and roots (S9 and S10 Tables) during the early stages of cold treatment (3 hr). The major categories were NAC-type TFs in shoots and WRKY-type TFs in roots. AP2/ERF-type TFs were uniquely expressed in shoots and roots and specific TFs were not shared between the two tissue types. In response to cold stress, AP2/ERF-type TFs represented the greatest number of DEGs in the shoots and roots of both cultivars. During recovery, bHLH-type TFs represented the major DEGs in the roots of both cultivars, although the expression of these genes was downregulated (Fig 8). The MYB and MYB-related TFs were the major upregulated TFs following cold recovery in the roots of TNG67. Several TFs were differentially expressed only during recovery in TNG67. In shoots, a GRAS TF (LOC_Os05g42130) was upregulated in TNG67 but downregulated in TCN1. bHLH (LOC_Os01g38610) and IAA20 (LOC_Os06g07040) were downregulated under cold stress but upregulated after recovery. In contrast, two AP2/ERF (LOC_Os01g21120 and LOC_Os02g52670) TFs and OsWRKY15 (LOC_Os01g46800) were upregulated in response to cold stress but downregulated after recovery. In roots, a TNG67-specific MYB TF (LOC_Os02g45080) was upregulated after recovery. In addition, AP2/ERF (LOC_Os01g64790) and HMG (LOC_Os02g44930) TFs were upregulated in response to cold stress but downregulated after recovery.

### Co-expression gene regulatory network

**Fig 9:**
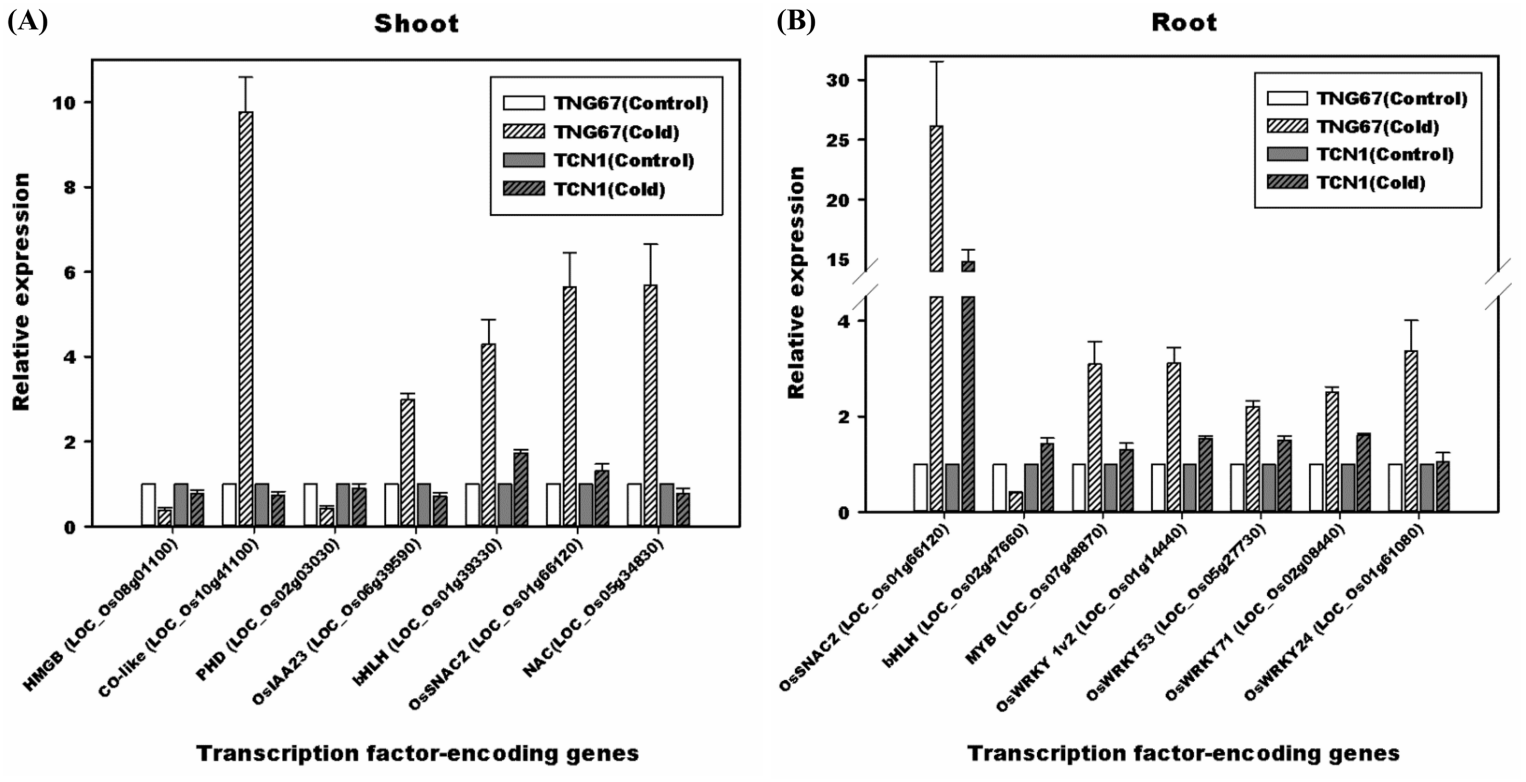
qRT-PCR confirmation of co-expressed genes related to cold tolerance based on the module results of a TF gene query, as predicted using Rice Oligonucleotide Array Database (http://www.ricearray.org/coexpression/coexpression.shtml).

Because the key DEGs detected, including 45 and 30 TFs in TNG67 shoots and roots, may represent crucial genes involved in cold stress tolerance in rice, we performed gene co-expression network analyses. We built a co-expression network with these TFs based on Rice Oligonucleotide Array Database (http://www.ricearray.org/coexpression/coexpression.shtml). Among the query genes, co-expressed genes related to abiotic stress with a correlation coefficient (r) cutoff of 0.8 were selected. A total of 45 TFs in shoots were inputted as query genes, generating 5 modules representing some TFs that were significantly linked to abiotic stress. An HMG-box DNA-binding protein (HMGB protein) (LOC_Os08g01100) and a CO-like protein that is orthologous to barley *HvCMF11* (LOC_Os10g41100) were found to be highly correlated with abiotic stress-related genes, and the PHD (LOC_Os02g03030) TF belonged to a module containing HMGB (LOC_Os08g01100) (S5 Fig). When we excluded these 3 genes in the 2 main modules and then re-analyzed the data, the 2 largest modules contained AUX/IAA (OsIAA23; LOC_Os06g39590), bHLH (LOC_Os01g39330), and 2 NAC TFs (SNAC2; LOC_Os01g66120 and LOC_Os05g34830) in the same module (S6 Fig). In addition, 4 modules were detected after inputting 30 root TFs as query genes. One module contained bHLH (LOC_Os02g47660; orthologous to AtbHLH063), and the other had NAC (SNAC2; LOC_Os01g66120). MYB (LOC_Os07g48870) was present in the smallest module. Interestingly, we found 4 WRKY TFs [OsWRKY 1v2 (LOC_Os01g14440); OsWRKY53 (LOC_Os05g27730); OsWRKY71 (LOC_Os02g08440) and OsWRKY24 (LOC_Os01g61080)] in the same module (S7 Fig). To confirm the specific species-, tissue- and cold-induced gene expression of these TFs, gene-specific primers were designed for the above-mentioned 13 genes, and qRT-PCR was conducted (Fig 9). The results obtained following exposure to low temperature stress for 3 hr were in accordance with those of module analysis, as shown in Fig 9. TFs that are known to be induced by cold, drought and submergence, including CO-like protein, OsIAA23, bHLH, SNAC2 and other NAC genes, were preferentially expressed in the shoots of TNG 67 but not in those of TCN1. In contrast, TFs such as SNAC2, MYB-containing DNA binding protein, OsWRKY1v2, OsWRKY53, OsWRKY71 and OSWRKY24, were identified in the roots of TNG67 but not in those of TCN1. A literature search revealed that SNAC2 is induced by drought, salinity, cold, wounding, and ABA treatment. Most importantly, the overexpression of SNAC2 in Zhonghua 11 2 (*japonica*) enhances its survival after severe cold stress (4–8°C for 5 days) [11]. These findings undoubtedly explain why the expression of this TF (SNAC2) was highly induced specifically in the shoots and roots of cold-tolerant TNG67 but not in those of TCN1. Moreover, OsIAA23-mediated auxin signaling has been shown to be crucial for the maintenance of the quiescent center in root tips (Jun et al., 2011). Additionally and interestingly, OsWRKY24, 51, 71, and 72 are induced by ABA and are involved in the ABA/GA balance in aleurone cells [35]. In the present study, we detected OsWRKY24 and 71 preferentially induced genes in TNG67 roots in response to cold stress, providing additional information regarding the role of ABA in rice cold stress tolerance, as has been shown in a previous physiological study. The other two WRKY TFs (OsWRKY 1v2 and OsWRKY53) were shown to be involved in salicylic acid and JA-mediated disease defense. OsWRKY 1v2 has ever been shown to be expressed at the early phase of chilling stress in *japonica* rice, while WRKY53 is expressed at the late stage (phase 2) which has been associated with disease resistance [36]. The expression patterns of these WRKY TFs may represent cross-talk occurring between abiotic and biotic stress responses. These TFs in the co-expression network may represent dominant roles in the regulation of the cold stress tolerance of TNG67.

## Discussion

Rice, particularly *indica* subspecies, is susceptible to chilling stress. Low temperatures can affect rice development during the germination, vegetative growth and reproductive stages. The screening of cold-tolerant rice varieties tends to be unsuccessful because of the poor correlation of cold resistance with different developmental periods [37]. In addition, cold stress tolerance is a quantitative trait that is determined by various quantitative trait loci. To understand the cold stress tolerance mechanisms used by rice to cope with cold sensitivity and to develop cold-tolerant rice cultivars, we conducted a comparative transcriptome study to identify changes in gene expression in response to cold and subsequent recovery between the roots and shoots of *japonica* (TNG67) and *indica* (TCN1) rice varieties. We aimed to discover some tolerance-related genes, important TFs and pathways that may benefit the breeding of potential rice varieties that are capable of withstanding cold.

### Differential expression in response to cold and during late recovery phases in TNG67 and TCN1

Comparative transcriptome analysis revealed more DEGs during the early cold response (3 hr, 4°C) in TNG67 than in TCN1 in both shoots and roots, with fewer DEGs in TNG67 than in TCN1 during the subsequent recovery phase (Fig 2). These results are consistent with those reported by Zhang et al. [14], who have shown that despite an intrinsic difference in constitutive cold tolerance-related gene expression in cold-resistant rice, the rapid and efficient reversion of gene expression during recovery remains an important factor in determining rice cold stress tolerance. Our results also indicate that TNG67 can reconfigure its genome for rapid gene expression and the re-establishment of homeostasis via metabolite adjustment when relieved of stress. Our gene clustering analysis clustered the DEGs into 3 distinct categories for the cold response and recovery phases (Fig 3). Genes that were specifically expressed in TNG67 during the early cold stress and recovery stages may represent cold tolerance-related genes that warrant further functional study.

### Efficient energy, nutrient resource conservation and recycling, water use and cell wall remodeling are essential for rice cold stress tolerance

Plants are sessile and unable to escape from environmental stresses. Thus, to acclimate, they must develop sensitive mechanisms for perceiving fluctuations in environmental cues and maintain homeostasis and the flexibility to adapt to environmental changes. Many stress conditions affect cellular ATP generation, protein synthesis and gene expression. The ability to ensure nutrient remobilization during energy deficiency by triggering the correct downstream transcriptional responses and metabolic adjustments is pivotal for plant survival.

TCN1 rice was found to be sensitive to low temperatures, as reflected by a reduced photosynthetic capacity and increased membrane leakage (Fig 1). Although GO:0015979 (photosynthesis) was significantly enriched in both TNG67 and TCN1, the number of genes assigned to this GO term in TCN1 was greater than that in TNG67. MapMan analysis also revealed enhanced gene expression associated with light reactions in TCN1 (Fig 4). In addition, the increased expression of mitochondrial electron transport–related genes was observed in TCN1 compared with TNG67. Under cold stress, insufficient activities of the light reaction in photosynthesis and the electron transport chain deprives the cell of ATP energy, which resulted in the accumulation of more ROS in TCN1 than in TNG67. However, MapMan biological pathway analysis showed that TNG67 increased the expression of glycolysis- and TCA cycle-related genes to avoid energy starvation during cold stress. Gene set enrichment analysis (GSEA) of roots showed that carbohydrate transport (GO:0008643) was enhanced under cold stress in TNG67 but not in TCN1. TNG67 may be able to change carbohydrate-coordinated partitioning from roots to shoots so that the shortage in the carbon supply due to diminished photosynthesis can be compensated for under cold stress. These results suggest that TNG67 possesses a more efficient mechanism to conserve energy and overcome the limited photosynthetic capacity under cold stress.

We also identified several over-represented GO terms, including GO:0019538 (protein metabolic process), GO:0006464 (protein modification process), GO:0016265 (death), GO:0006950 (response to stress) and GO:0016265 (cell death), in TNG67 during the early stage of cold stress. GO:0016265 (death) was significantly repressed in TCN1 in response to 24 hr of cold stress. Translational regulation at the protein level and apoptosis or PCD may be important for rice cold stress tolerance, especially the protein modification process (GO:0006464). Previous reports have demonstrated a close association between PCD-related genes and increased cold stress tolerance in *japonica* rice with introgression of an *indica* allele [13]. Cell death may be considered as not only simply a damaging consequence of cold stress but also part of a regulated process that permits flexibility in the cold stress response. Cell death is also an important physiological process for the removal of senescent and damaged cells during abiotic stress [38]. Apoptosis (GO:0006915) may act as a signal for TNG67 under cold stress and trigger another set of genes associated with GO:0006950 (response to stress) to initiate cold tolerance mechanisms. In addition, the regulation of apoptosis is highly associated with the ubiquitin conjugation system [39]. We found that in response to cold treatment, the expression of genes related to E2, E3 ligase, and the 20S proteasome were highly induced in TNG67 but not in TCN1 (S2 Fig). Under stress, protein translation can be arrested, and protein levels can be regulated via a proteasome-dependent degradation process [40]. Apoptosis and ubiquitin may play roles in metabolic reprogramming by recycling or reutilizing degraded substrates for the *de novo* synthesis of proteins to increase cold stress tolerance.

We identified three genes assigned to GO:0006950 (response to stress), LOC_Os04g16450, LOC_Os05g14240 and LOC_Os06g22960, that were predicted to be similar to aquaporin proteins and were induced in response to cold stress in both TNG67 and TCN1, but predominantly in TNG67 roots. Previous studies have demonstrated that under cold stress, the ability to control water absorption in roots is a more important characteristic to overcome stress-related damage than the regulation of transpiration in the leaf [41]. Our MapMan analysis revealed a tendency toward the increased synthesis of phenylpropanoid and lignin in the roots of TNG67 compared with those of TCN1 under cold stress. Cell wall remodeling was found to be a long-term adaption mechanism for plants to cope with multiple biotic or abiotic stresses [42]. Whether this is also true for cold stress tolerance in TNG67 remains to be determined in future studies.

### ABA, polyamine, auxin and JA hormone pathways act together or alone to regulate cold-responsive TFs and cold tolerance-related gene expression in rice

Recent findings have demonstrated the importance of phytohormone cross-talk in the responses and resistance of plants to multiple abiotic stresses [24]. In particular, the integration or coordination of hormone-related gene expression under different abiotic stress conditions has become essential to the understanding of plant abiotic stress tolerance mechanisms. Despite discrepancies in reported levels of regulation, from mRNAs to proteins and even metabolites, global transcriptome analysis of the expression, pathways and modules of co-expressed genes still represents a powerful approach for dissecting cross-talk among plant hormones during environmental stress.

In previous studies, Lee et al. [5] have demonstrated an accelerated increase in the concentration of ABA in TNG67 within 4 hr of cold treatment. The expression of ABA biosynthetic genes, including LOC_Os09g38320 (PSY3), LOC_Os01g1271tabolits0 (SDR), LOC_Os12g42280 (NCED2) and LOC_Os01g03750 (ABA4), was higher in the shoots of TNG67 compared with those of TCN1 and may explain why TNG67 had a higher ABA level during cold stress. Among these genes, LOC_Os12g42280 (NCED2) responded more strongly to cold stress in the roots of TNG67 than in those of TCN1 after 3 hr of exposure, and this effect was enhanced at 24 hr. This gene is a key contributor to ABA biosynthesis in TNG67 and TCN1 roots. The roots may represent the major location of the synthesis of ABA, which is transported to shoots during cold stress. When ABA is applied exogenously to plants, an immediate increase in its levels is observed in leaves [43]. However, ABA levels do not increase significantly until leaf turgor has dropped to zero, despite the presence of drying soil. Under such conditions, plants that exhibit stomatal closure possess an efficient system for the transport of ABA from root to guard cells through the xylem [44]. Furthermore, genes related to ABA signal transduction pathways were preferentially expressed in TNG67 shoots. During cold stress, the expression levels of the 2 ABA-GTase genes LOC_Os07g32010 and LOC_Os07g32060 were significantly decreased in TNG67 shoots; therefore, the increased ABA level may have been a result of the inactivation of ABA-GTase to switch ABA from a storage to a free form. Furthermore, the expression of LOC_Os02g47470 (ABA8OX1) and LOC_Os09g28390 (ABA8OX3), which are responsible for the deactivation of ABA, was induced to a greater extent in shoots compared with roots. In maize, the ABA catabolism rate increases by 11-fold in response to water stress. Pharmacological experiments have shown that this phenomenon may be beneficial for *de novo* ABA synthesis in roots and critical for rapid early adaptation when there is a requirement for ABA. These studies have revealed an important role of ABA in cold stress tolerance in TNG67. The role of roots in ABA-mediated cold tolerance and the mechanism(s) underlying the long distance root-to-shoot transport of ABA require further clarification.

The gene expression pattern revealed by our microarray analysis is inconsistent with that of a previous study investigating ACC and ET production in response to cold stress [4]. A recent study has reported that ET is involved in the transcriptional regulation of CBF regulons and negatively affects freezing stress tolerance in *Arabidopsis* [45]. This observation may aid in explaining our findings. As an alternative explanation, there may have been competition between polyamines and ET for SAM as a common substrate to produce methylthioadenosine. SAM activity shifts toward the polyamine biosynthesis pathway to promote the accumulation of polyamines under cold stress. These genes must be synthesized in advance during cold stress so that the corresponding gene products are quickly translated after removal of plants from the cold. In TNG67, the ACC and ET levels increased during recovery. However, in TCN1, even the ACC level increased during recovery, and it could not be converted into ET. The gradual increase in the ET level in response to cold stress, which reverted after recovery, may represent an important index for evaluating the capacity of cold stress tolerance in rice [4]. The microarray data further showed that ET biosynthesis genes, such as LOC_Os09g27820 (ACO1), LOC_Os05g05680 (ACO5) and LOC_Os05g05670 (ACO6), which are responsible for converting ACC to ET, were upregulated in TNG67 roots at low temperatures and displayed prolonged expression during recovery. Ethylene exerts an antagonistic function against ABA, which further supports its role in detecting the disappearance of stress in rice plants.

Under cold stress, ornithine decarboxylase (ODC) genes were repressed, but ADC genes were induced. These results indicated that the Put biosynthesis pathways were shifted in the direction of ADC. In *Arabidopsis*, polyamine biosynthesis requires the activity of the rate-limiting enzyme ADC to synthesize Put [46], a key molecule that contributes to cold tolerance in *Arabidopsis* [47]. ADC genes, including LOC_Os06g04070 (ADC) and LOC_Os08g33620|LOC_Os06g04070 (ADC), were preferentially induced in TNG67 compared with TCN1 in both shoots and roots. The transcriptional regulation of ADC corresponds to its activity and the Put level, which increased rapidly in TNG67; however, ODC activity remained unchanged after the cold treatment [6]. The promoter sequence of *OsADC1* was found to contain a CRT/DRE element, which may mediate early and transient increases in *OsADC1* expression in response to cold stress. In addition, the *OsADC2* promoter contains 5 ABA-responsive elements that can increase *OsADC2* expression after the *de novo* biosynthesis of ABA [48]. At low temperatures, Put interferes with ABA biosynthesis and gene expression and affects the ABA level [27]. The free spermidine: spermine levels and the activity of SAMDC are increased in chilled TNG67 [6]. We found that SAMDC-encoding genes, including LOC_Os02g39790 and LOC_Os04g42090, were induced in TNG67 compared with TCN1, providing more aminopropyl donors for spermidine synthase (SPDS) or spermine synthase (SPMS), resulting in higher levels of free spermidine: spermine. Cross-talk among polyamines, ABA and ET coordinately establish the state of rice cold stress tolerance.

GA biosynthesis-related genes, including LOC_Os06g02019 (KAO), LOC_Os03g63970 (GA20ox1), and LOC_Os01g66100 (GA20ox2), were suppressed under cold, and LOC_Os01g66100 (GA20ox2) expression was higher in TNG67 than in TCN1 in both shoots and roots. In contrast, deactivated genes in shoots, including LOC_Os01g55240 (GA2ox3) and LOC_Os04g44150 (GA2ox6), were upregulated to a greater extent in TNG67 than in TCN1. Overall, GA seems to decrease the ABA level during cold stress, which is consistent with the antagonistic activity that has been reported between their levels in response to cold temperatures. In bromegrass, enhanced cold tolerance due to exogenous ABA is eliminated by exogenous GA [49]. GA exerts its physiological function by degrading its receptor, DELLA. Under salinity and water stress, the endogenous accumulation of ABA and ET stimulates DELLA gene expression and leads to DELLA-dependent growth inhibition [24]. This well-illustrated example integrating ABA, ET and GA at the level of DELLA may provide insights regarding rice cold stress tolerance.

Predicting changes in the CK level in response to cold stress is difficult because the expression of the genes responsible for its biosynthesis and degradation were downregulated and upregulated simultaneously. However, the majority of genes related to the CK signal transduction pathway were more strongly repressed in TNG67 than in TCN1; for example, LOC_Os04g36070 (RR1) and LOC_Os07g26720 (RR7) in shoots and LOC_Os03g50860 (HK4), LOC_Os02g58350 (RR3), LOC_Os01g72330 (RR4) and LOC_Os07g26720 (RR7) in roots. AHK3 is a cold-inducible A-type *Arabidopsis* response regulator (ARR) that is involved in cold signaling. The phenotype of *arr7 Arabidopsis* mutants includes tolerance to freezing [31]. AHK2, AHK3, and AHK4 function as negative regulators of ABA signaling and osmotic stress signaling in both ABA-dependent and-independent pathways [50]. Cytokine oxidase (CKX) transgenic *Arabidopsis* plants with CK deficiency show increased sensitivity to ABA and enhanced tolerances to drought and salt stresses [30]. It will be interesting to further assess whether TNG67 achieves cold stress tolerance by repressing CK signaling and increasing sensitivity to ABA.

The expression of the JA biosynthesis genes LOC_Os08g04800 (*OsDAD1; 2*), LOC_Os12g37260 (*OsLOX2; 2*), and LOC_Os08g35740 (*OsOPR7*) was upregulated in the shoots, and that of LOC_Os03g12500 (*OsAOS1*) was upregulated in the roots of TNG67. The JA level may be increased to a greater extent in TNG67 than in TCN1 in response to cold stress. A previous study has shown that the addition of MeJa to TCN1 alleviates injury due to cold [7]. A study of a cold-tolerant introgressed line, K354, has shown that enhanced cold resistance is associated with *OsFAD7* upregulation [13]. In *Arabidopsis*, At*FAD7* expression is induced locally by wounding, leading to the rapid and sustainable accumulation of JA [51]. Our preliminary study also revealed *OsFAD7* induction in TNG67 in response to cold stress. Additionally, the ratio of dienoic and trienoic fatty acids to total fatty acids was higher in TNG67. TNG67 is potentially able to synthesize JA quickly under cold stress. Jasmonates may also induce intracellular alkalinization and guard cell closure [52, 53]. In *Arabidopsis*, ABA may participate in cross-talk with MeJA signal transduction to close stomata. Furthermore, MeJA may stimulate the expression of *AtNCED3*, a crucial enzyme for ABA biosynthesis [54]. Theoretically, if TNG67 accumulates JA faster than TCN, it should be able to synthesize more ABA, which may explain why TNG67 was able to assimilate more ABA than TCN1 at low temperatures and was resistant to cold stress. Recently, jasmonate has been shown to play a crucial role as an upstream signal in the *ICE-CBF/DREB1* pathway to increase *Arabidopsis* freezing tolerance [34]. Indeed, our microarray data showed that *OsCBF1* (LOC_Os08g31580) was more strongly induced in TNG67 than in TCN1 in both shoots and roots. Furthermore, downstream *RAP2*.*6-like* (LOC_Os09g28440) was induced in the roots of TNG67 but was repressed in those of TCN1 after 3 hr of cold treatment. In addition, *ZAT10-like* (LOC_Os12g39400) was repressed in TCN1 roots. Thus, TNG67, but not TCN1, may transcriptionally activate JA biosynthesis-related genes and enhance gene expressions in the ICE-CBF/DREB1 regulon in response to cold stress.

Auxin has been established as a plant hormone that regulates growth and development. The role of auxin in abiotic stress has been gradually elucidated. In *Arabidopsis*, inflorescence gravitropism is inhibited by cold stress via auxin regulation [55]. Cold stress can block the asymmetric redistribution and intracellular cycling of PIN3 and inhibit the intracellular cycling of PIN2. In *Arabidopsis* roots, cold stress may reduce shootward auxin transport and alter the intracellular auxin gradient [56]. Therefore, this gradient may exert effects on plant growth and development to determine cold tolerance [32]. According to our microarray data, the expression levels of auxin biosynthesis and signaling-related genes were preferentially induced in TNG67. This finding may indicate the involvement of auxin in the cold tolerance capacity of TNG67.

Low temperatures can alter the gene expression profiles of many hormones, and the complicated interaction or cross-talk among different hormones can act coordinately or alone to regulate the mechanisms underlying cold stress tolerance in rice. Among these phytohormones, ABA, polyamines and JA are the major determinants mediating the capacity for cold tolerance of TNG67.

### Potential roles of genotype-dependent and tissue-specific TFs, especially NACs and WRKYs, in rice cold stress tolerance

Recently, NAC proteins have been found to be involved in plant responses to pathogens and environmental stimuli. In the present study, NAC TFs were the predominant cold stress–induced genes detected during the early stages of cold stress in rice. For example, *OsNAC6/SNAC2* (LOC_Os01g66120) was induced after 3 hr of cold treatment in the shoots and roots of TNG67 compared with those of TCN1. *OsNAC6/SNAC2* expression is induced by cold, drought, salinity stress, JA, wounding and blast disease [11, 57]. In addition, we identified another *OsNAC3* (LOC_Os07g12340) as a drought-induced gene, and transgenic rice overexpressing this gene have been reported with improved dehydration tolerance [58]. Thus, *OsNAC3* may participate in cross-talk during cold and drought stresses.

The C2H2-type Zn-finger TF *OsZOS11-10* (LOC_Os11g47630) was highly induced in TNG67 shoots. This result is consistent with previous reports showing that *OsZOS11-10* is highly induced by cold stress similar to *OsDREB1* and is negatively regulated by *OsDREB1B* [59]. The lack of an immediate negative effect of *OsZOS11-10* on *OsDREB1* expression may be explained by competition with other TFs or regulation at the post-transcriptional or translational level.

Three CO-like TFs [LOC_Os06g16370 (the same as *Hd1*), LOC_Os08g15050, and LOC_Os10g41100) were induced in TNG67 shoots in response to cold stress. CO-like TFs are central regulators in the photoperiod pathway [60]. In addition, the expression of *MaCOL1*, a CONSTANS-like gene in banana fruit, is significantly enhanced by abiotic and biotic stresses, such as chilling and pathogen infection [61]. In addition, the peak transcript levels of *DREB1/CBF* genes are high and low after the exposure of plants to low temperatures following subjective dawn and evening, respectively. These results indicate that the cold induction of *DREB1/CBF* genes is circadian-gated [62]. Further, LOC_Os06g16370 and LOC_Os10g41100 were highly expressed under cold stress compared with other TFs and may be important for rice cold stress tolerance.

An R2R3-type MYB gene, OsMYB2 (LOC_Os03g20090), is a transactivator that is involved in salt, cold, and dehydration tolerance in rice. *OsMYB2* overexpression in rice enhances the expression of genes encoding proline synthase and transporters [63]. The relative gene expression levels of proline synthase and transporters did not differ significantly between TNG67 and TCN1. However, OsMYB2 was uniquely upregulated in TNG67 roots during the early stages of cold stress in our study. Further experiments are needed to confirm the expression of proline-related genes and accumulation of proline in TNG67 and TCN1 roots.

Six genes in TNG67 and TCN1 were expressed in both shoots and roots, including LOC_Os01g53220, a heat shock factor that has been reported to be upregulated in rice cultivars that are resistant to the brown planthopper [64]. This heat shock factor may play a role in the interaction between abiotic and biotic stresses in rice because cross-talk between the abiotic and biotic stress responses has been increasingly reported in recent years.

To clarify the relationship between hormones and cold-induced TFs, we further analyzed the expression of these genes using RiceXPro database (http://ricexpro.dna.affrc.go.jp/) (S9 and S10 Tables). After 3 hr of cold treatment, approximately 85% to 60% of TF expression in roots was mainly affected by JA, ABA and auxin (IAA), whereas only 40% to 25% of TF expression in shoots was affected by these hormones (S8 Fig). Gibberellin and BR had almost no effect. Auxin may partially contribute to these effects by coordinating with ABA and JA in roots to regulate these candidate TFs (S10 Table).

### Genes involved in cytokine signal transduction may be important for re-establishing homeostasis during recovery from cold stress

We found that the GO Slim category GO:0000160 [two-component signal transduction system (phosphorelay)] was specifically enriched in the shoots of TNG67 but not in those of TCN1 after recovery from cold exposure. Genes in this category are all OsRR type-A genes, including OsRR1 (LOC_Os04g36070), OsRR2 (LOC_Os02g35180), OsRR4 (LOC_Os01g72330), OsRR6 (LOC_Os04g57720) and OsRR10 (LOC_Os11g04720) (Fig 6). The expression of type-A ARR genes is known to be induced by cytokines [65]. Indeed, in our study, the LOC_Os05g47840 (IPT7) gene that encodes the rate-limiting enzyme in cytokine biosynthesis was up-regulated in the roots of TNG67 but not in those of TCN1 during the recovery stage. Moreover, in *Arabidopsis*, the *arr5*, *arr6*, and *arr7* mutants displayed elevated cold tolerance, suggesting that type-A ARRs function as negative regulators in response to cold stress [31]. In response to gradual relief from cold stress, the cold response of TNG67 may be suppressed in shoots through an increase in IPT7 gene expression during the recovery stage. Moreover, CK can increase the activities of the ET biosynthesis genes ACS4 [66] and ACS5 [67] via a post-transcriptional mechanism. These results further indicate that the increased ET level observed in TNG67 during the recovery stage may have been regulated at the post-transcriptional level. We also found that GO:0015977 (carbon fixation), GO:0006457 (protein folding), GO:0008643 (carbohydrate transport) and GO:0006952 (defense response) were specifically enriched after 24 hr of recovery in the roots of TNG67 but not in those of TCN1. These results indicate that roots may play an important role during the recovery stage in response to cold stress.

## Conclusions

**Fig 10:**
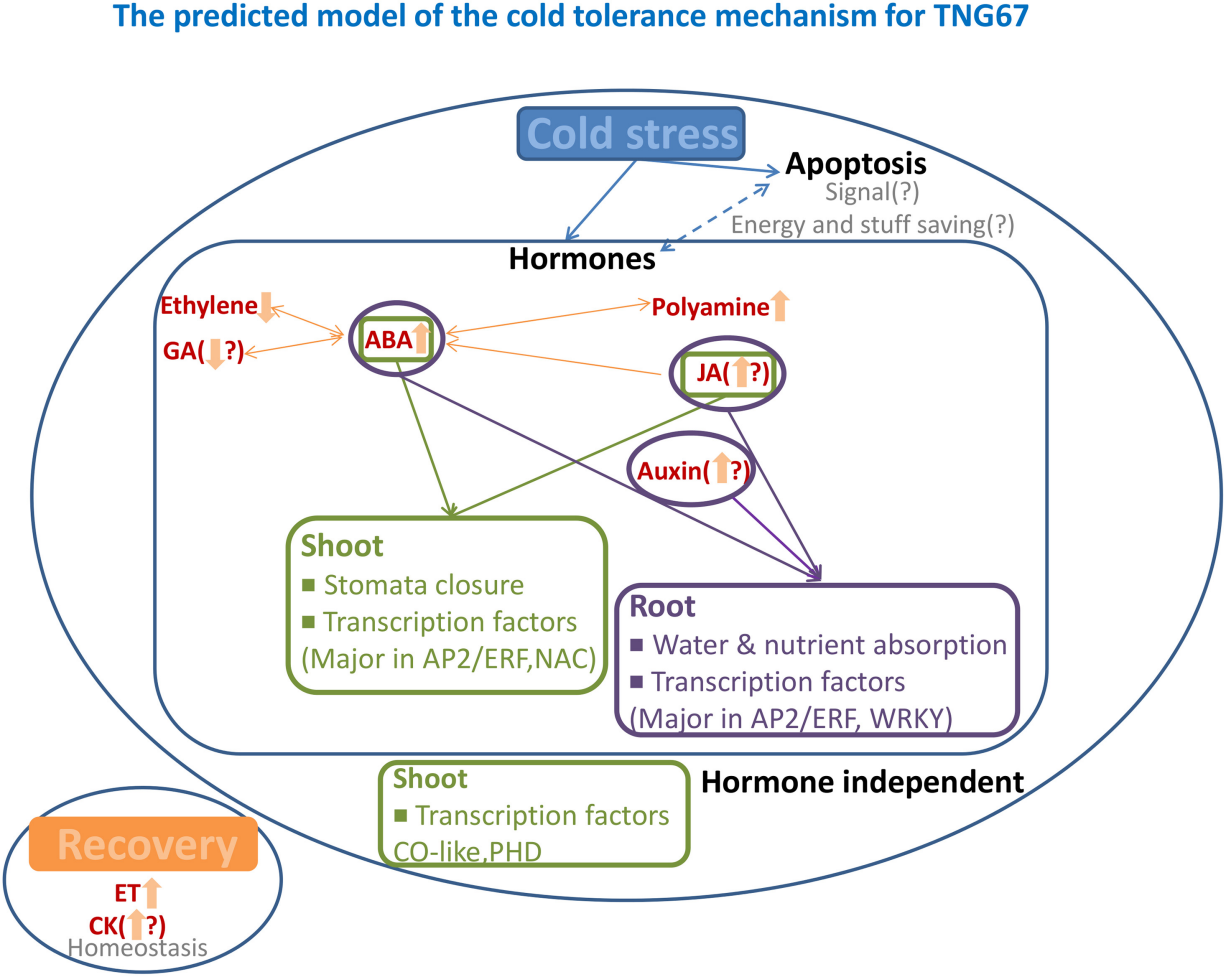
A putative model for the cold stress-tolerance mechanism in TNG67.

Our transcriptome study revealed a preliminary model for the resistance of TNG67 to cold stress (Fig 10). Following exposure to cold, the rapid induction of ABA and JA in TNG67 accelerated stomatal closure to rapidly prevent water loss. These 2 hormones may interact with one another to alter the expression of a specific set of TFs in shoots (mainly NACs). In roots, auxin may participate in cross-talk with these 2 hormones to adjust the expression of other sets of TFs, such as WRKYs, to promote cold stress tolerance in rice. Polyamine is another key component of cold tolerance in TNG67. In response to cold stress, the level of ET was reduced and then elevated after recovery in these seedlings. Ethylene may facilitate the detection of stress relief because it has an antagonistic function to ABA. CK may also be involved in this process through interacting with ET. Additionally, the activation of apoptosis-related genes may be an important signal to trigger cold stress tolerance. This process can eliminate redundant or harmful cells and recycle the constituents of cells, and it is probably a more energy-efficient means of responding to environmental stimuli. In TCN1 under cold stress, the expression of photosynthesis-related genes increased as a consequence of energy starvation. However, this process causes ROS accumulation and leads to oxidative stress with cold-induced water loss. In contrast, the expression levels of TCA cycle- and glycolysis-related genes were greater in TNG67 than in TCN1. TNG67 may compensate for decreased energy production without excessively increasing photosynthetic reactions to avoid oxidative stress. Our study expands upon the current understanding of cold stress tolerance in rice, and our findings may be used to facilitate the breeding of cold-tolerant rice.

## Supporting Information

S1 Fig Correlation of changes in gene expression between microarray analysis and quantitative PCR. (PDF)

S2 Fig MapMan analysis of genes involved in ubiquitin-dependent degradation in shoots exposed to cold for (A) 3 hr and (B) 24 hr (C) and allowed to recover for 24 hr after cold treatment. (PDF)

S3 Fig GUS staining of DR5 rice seedlings following cold stress and recovery. (PDF)

S4 Fig Treatment of TNG67 and TCN1 rice seedlings exposed to 24 hr of cold stress (4°C) with jasmonic acid (JA) inhibitor (IBU). (PDF)

S5 Fig Co-expressed gene network of transcription factor (TF)-encoding DEGs in TNG67 shoots in response to 3 hr of cold stress. (TIF)

S6 Fig Co-expressed gene network of TF-encoding DEGs in TNG67 shoots in response to 3 hr of cold stress after excluding the 3 genes in the 2 main modules shown in S5 Fig. (TIF)

S7 Fig Co-expressed gene network of TF-encoding DEGs in TNG67 roots in response to 3 hr of cold stress. (TIF)

S8 Fig The ratio of TF-encoding DEGs in TNG67 with P-values of greater than < 0.01 and significantly altered expression levels in the shoots of TNG67 compared with those of TCN1 in response to 3 hr of cold treatment and hormones, as predicted using RiceXPro database. (PDF)

S1 Table List of gene-specific primers used for quantitative RT-PCR. (PDF)

S2 Table DEGs expressed under cold and recovery conditions in shoots of TNG 67 and TCN1. (XLSX)

S3 Table DEGs expressed under cold and recovery conditions in roots of TNG 67 and TCN1. (XLSX)

S4 Table GSEA results for the biological process category, showing significant enrichment (p≤0.05) in TNG67 and TCN1 shoots in response to cold stress and recovery. (XLSX)

S5 Table GSEA results for the biological process category, showing significant enrichment (p≤0.05) in TNG67 and TCN1 roots in response to cold stress and recovery. (XLSX)

S6 Table IDs/descriptions of genes encoding proteins involved in the light reaction, mitochondrial electron transport, the TCA cycle, and glycolysis pathways and proteasome, phenylpropanoid and lignin-related DEGs in response to cold stress and recovery, as analyzed with MapMan software. (XLS)

S7 Table IDs/descriptions of DEGs-coded TFs in response to cold stress and recovery in shoots of TNG67 and TCN1. (XLSX)

S8 Table IDs/descriptions of DEGs-coded TFs in response to cold stress and recovery in roots of TNG67 and TCN1. (XLSX)

S9 Table The effects of various phytohormones on TFs preferentially expressed in the shoots (roots) of TNG67 during the early stages of cold stress (3 hr), as predicted using RiceXPro database (p < 0.01). A heat map of TF-encoding DEGs constructed based on the microarray data is shown. In this table, “S” represents shoot and “R” represents root. Genes that were induced or repressed by a given hormone treatment listed in RiceXPro database are denoted by “**↑**” and “**↓**”, respectively. “N” indicates that no data are available in RiceXPro database for these TFs. “-” indicates no effect in the presence of specific plant hormones.
(PDF)

S10 Table The effects of various phytohormones on TFs preferentially expressed in the shoots (roots) of TNG67 during the early stages of cold stress (3 hr), as predicted using RiceXPro database (p < 0.01). A heat map of TF-encoding DEGs constructed based on the microarray data is shown. In this table, “S” represents shoot and “R” indicates root. Genes that were induced or repressed by a given hormone treatment listed in RiceXPro database are denoted by “**↑**” and “**↓**”, respectively. “N” indicates that no data are available in RiceXPro database for these TFs. “-” indicates no effect in the presence of specific plant hormones.
(PDF)

## References

[1] Baena-González E. Energy signaling in the regulation of gene expression during stress. Molecular Plant. 2010;3(2):300–13. 10.1093/mp/ssp113 20080814

[2] Arbona V, Manzi M, Ollas C, Gómez-Cadenas A. Metabolomics as a tool to investigate abiotic stress tolerance in plants. Int J Mol Sci. 2013;14(3):4885–911. 10.3390/ijms14034885 23455464 PMC3634444

[3] Peleg Z, Blumwald E. Hormone balance and abiotic stress tolerance in crop plants. Curr Opin Plant Biol. 2011;14(3):290–5. 10.1016/j.pbi.2011.02.001 21377404

[4] Lee T-M, Chu C. Regulation of chilling tolerance in rice seedlings by plant hormones. Korean J Crop Sci. 1992;37:288–98.

[5] Lee TM, Lur HS, Chu C. Role of abscisic acid in chilling tolerance of rice (Oryza sativa L.) seedlings. I. Endogenous abscisic acid levels. Plant, Cell Environ. 1993;16(5):481–90. 10.1111/j.1365-3040.1993.tb00895.x

[6] Lee T-M, Lur H-S, Chu C. Role of abscisic acid in chilling tolerance of rice (Oryza sativa L.) seedlings.: II. Modulation of free polyamine levels. Plant Sci. 1997;126(1):1–10.

[7] Lee TM, Lur HS, Lin YH, Chu C. Physiological and biochemical changes related to methyl jasmonate-induced chilling tolerance of rice (*Oryza sativa* L.) seedlings. Plant, Cell Environ. 1996;19(1):65–74. 10.1111/j.1365-3040.1996.tb00227.x

[8] Chinnusamy V, Zhu J, Zhu J-K. Cold stress regulation of gene expression in plants. Trends Plant Sci. 2007;12(10):444–51. 17855156 10.1016/j.tplants.2007.07.002

[9] Vannini C, Locatelli F, Bracale M, Magnani E, Marsoni M, Osnato M, et al Overexpression of the rice *Osmyb4* gene increases chilling and freezing tolerance of *Arabidopsis thaliana* plants. Plant J. 2004;37(1):115–27. 10.1046/j.1365-313X.2003.01938.x 14675437

[10] Dai X, Xu Y, Ma Q, Xu W, Wang T, Xue Y, et al Overexpression of an *R1R2R3 MYB* Gene, *OsMYB3R-2*, increases tolerance to freezing, drought, and salt stress in transgenic *Arabidopsis* . Plant Physiol. 2007;143(4):1739–51. 10.1104/pp.106.094532 17293435 PMC1851822

[11] Hu H, You J, Fang Y, Zhu X, Qi Z, Xiong L. Characterization of transcription factor gene SNAC2 conferring cold and salt tolerance in rice. Plant Mol Biol. 2008;67(1–2):169–81. 10.1007/s11103-008-9309-5 18273684

[12] Rabbani MA, Maruyama K, Abe H, Khan MA, Katsura K, Ito Y, et al Monitoring expression profiles of rice genes under cold, drought, and high-salinity stresses and abscisic acid application using cDNA microarray and RNA Gel-Blot analyses. Plant Physiol. 2003;133(4):1755–67. 10.1104/pp.103.025742 14645724 PMC300730

[13] Zhang F, Huang L, Wang W, Zhao X, Zhu L, Fu B, et al Genome-wide gene expression profiling of introgressed indica rice alleles associated with seedling cold tolerance improvement in a japonica rice background. BMC Genomics. 2012;13(1):461 10.1186/1471-2164-13-461 22953761 PMC3526417

[14] Zhang T, Zhao X, Wang W, Pan Y, Huang L, Liu X, et al Comparative transcriptome profiling of chilling stress responsiveness in two contrasting rice genotypes. PLoS One. 2012;7(8):e43274 10.1371/journal.pone.0043274 22912843 PMC3422246

[15] Chawade A, Lindlöf A, Olsson B, Olsson O. Global expression profiling of low temperature induced genes in the chilling tolerant japonica rice Jumli Marshi. PLoS One. 2013;8(12):e81729 10.1371/journal.pone.0081729 24349120 PMC3861252

[16] Cheng C, Yun K-Y, Ressom HW, Mohanty B, Bajic VB, Jia Y, et al An early response regulatory cluster induced by low temperature and hydrogen peroxide in seedlings of chilling-tolerant japonica rice. BMC Genomics. 2007;8(1):175 10.1186/1471-2164-8-175 17577400 PMC1925099

[17] Yun K-Y, Park M, Mohanty B, Herath V, Xu F, Mauleon R, et al Transcriptional regulatory network triggered by oxidative signals configures the early response mechanisms of japonica rice to chilling stress. BMC Plant Biol. 2010;10(1):1–29. 10.1186/1471-2229-10-16 20100339 PMC2826336

[18] Fennell A, Markhart AH. Rapid acclimation of root hydraulic conductivity to low temperature. J Exp Bot. 1998;49(322):879–84. 10.1093/jxb/49.322.879

[19] Ahamed A, Murai-Hatano M, Ishikawa-Sakurai J, Hayashi H, Kawamura Y, Uemura M. Cold stress-induced acclimation in rice is mediated by root-specific aquaporins. Plant and Cell Physiol. 2012;53(8):1445–56. 10.1093/pcp/pcs089 22711693

[20] Narsai R, Castleden I, Whelan J. Common and distinct organ and stress responsive transcriptomic patterns in *Oryza sativa* and *Arabidopsis thaliana* . BMC Plant Biol. 2010;10(1):262 10.1186/1471-2229-10-262 21106056 PMC3095337

[21] Chen J-S, Lin S-C, Chen C-Y, Hsieh Y-T, Pai P-H, Chen L-K, et al Development of a microarray for two rice subspecies: characterization and validation of gene expression in rice tissues. BMC Research Notes. 2014;7(15):1–9. 24398116 10.1186/1756-0500-7-15 PMC3891988

[22] Thimm O, Bläsing O, Gibon Y, Nagel A, Meyer S, Krüger P, et al MAPMAN: a user-driven tool to display genomics data sets onto diagrams of metabolic pathways and other biological processes. Plant J. 2004;37(6):914–39. 10.1111/j.1365-313X.2004.02016.x 14996223

[23] Jung K-H, An G. Application of MapMan and RiceNet drives systematic analyses of the early heat stress transcriptome in rice seedlings. Journal of Plant Biology. 2013;55(6):436–49. 10.1007/s12374-012-0270-0

[24] Kohli A, Sreenivasulu N, Lakshmanan P, Kumar P. The phytohormone crosstalk paradigm takes center stage in understanding how plants respond to abiotic stresses. Plant Cell Rep. 2013;32(7):945–57. 10.1007/s00299-013-1461-y 23749097

[25] Welsch R, Wust F, Bar C, Al-Babili S, Beyer P. A third phytoene synthase is devoted to abiotic stress-induced abscisic acid formation in rice and defines functional diversification of phytoene synthase genes. Plant Physiol. 2008;147(1):367–80. 10.1104/pp.108.117028 18326788 PMC2330301

[26] Gill SS, Tuteja N. Reactive oxygen species and antioxidant machinery in abiotic stress tolerance in crop plants. Plant Physiol Biochem. 2010;48(12):909–30. 10.1016/j.plaphy.2010.08.016 20870416

[27] Cuevas JC, Lopez-Cobollo R, Alcazar R, Zarza X, Koncz C, Altabella T, et al Putrescine is involved in Arabidopsis freezing tolerance and cold acclimation by regulating abscisic acid levels in response to low temperature. Plant Physiol. 2008;148(2):1094–105. 10.1104/pp.108.122945 18701673 PMC2556839

[28] Magome H, Yamaguchi S, Hanada A, Kamiya Y, Oda K. *dwarf and delayed-flowering 1*, a novel *Arabidopsis* mutant deficient in gibberellin biosynthesis because of overexpression of a putative AP2 transcription factor. Plant J. 2004;37(5):720–9. 10.1111/j.1365-313X.2003.01998.x 14871311

[29] Achard P, Cheng H, De Grauwe L, Decat J, Schoutteten H, Moritz T, et al Integration of plant responses to environmentally activated phytohormonal signals. Science. 2006;311(5757):91–4. 10.1126/science.1118642 16400150

[30] Nishiyama R, Watanabe Y, Fujita Y, Le DT, Kojima M, Werner T, et al Analysis of cytokinin mutants and regulation of cytokinin metabolic genes reveals important regulatory roles of cytokinins in drought, salt and abscisic acid responses, and abscisic acid biosynthesis. Plant Cell. 2011;23(6):2169–83. 10.1105/tpc.111.087395 21719693 PMC3160038

[31] Jeon J, Kim NY, Kim S, Kang NY, Novák O, Ku S-J, et al A subset of cytokinin two-component signaling system plays a role in cold temperature stress response in *Arabidopsis* . J Biol Chem. 2010;285(30):23371–86. 10.1074/jbc.M109.096644 20463025 PMC2906329

[32] Rahman A. Auxin: a regulator of cold stress response. Physiol Plant. 2013;147(1):28–35. 10.1111/j.1399-3054.2012.01617.x 22435366

[33] Du H, Liu H, Xiong L. Endogenous auxin and jasmonic acid levels are differentially modulated by abiotic stresses in rice. Front Plant Sci. 2013;4 10.3389/fpls.2013.00397 PMC3793129 24130566

[34] Hu Y, Jiang L, Wang F, Yu D. Jasmonate regulates the INDUCER OF CBF EXPRESSION–C-REPEAT BINDING FACTOR/DRE BINDING FACTOR1 cascade and freezing tolerance in *Arabidopsis* . Plant Cell. 2013 10.1105/tpc.113.112631 PMC3784588 23933884

[35] Xie Z, Zhang Z-L, Zou X, Huang J, Ruas P, Thompson D, et al Annotations and functional analyses of the rice WRKY gene superfamily reveal positive and negative regulators of abscisic acid signaling in aleurone cells. Plant Physiol. 2005;137(1):176–89. 10.1104/pp.104.054312 15618416 PMC548849

[36] Yun K-Y, Park MR, Mohanty B, Herath V, Xu F, Mauleon R, et al Transcriptional regulatory network triggered by oxidative signals configures the early response mechanisms of japonica rice to chilling stress. BMC Plant Biol. 2010;10(1):16 10.1186/1471-2229-10-16 20100339 PMC2826336

[37] Cruz RPd, Sperotto RA, Cargnelutti D, Adamski JM, de FreitasTerra T, Fett JP. Avoiding damage and achieving cold tolerance in rice plants. Food and Energy Security. 2013;2(2):96–119. 10.1002/fes3.25

[38] Palavan-Unsal N, Buyuktuncer E-D, Tufekci MA. Programmed cell death in plants. J Cell Mol Biol. 2005;4:9–23.

[39] Broemer M, Meier P. Ubiquitin-mediated regulation of apoptosis. Trends Cell Biol. 2009;19(3):130–40. 10.1016/j.tcb.2009.01.004 19217783

[40] Kurepa J, Wang S, Li Y, Smalle J. Proteasome regulation, plant growth and stress tolerance. Plant Signal Behav. 2009;4(10):924–7. 19826220 10.4161/psb.4.10.9469 PMC2801354

[41] Aroca R, Tognoni F, Irigoyen JJ, Sanchez-Diaz M, Pardossi A. Different root low temperature response of two maize genotypes differing in chilling sensitivity. Plant Physiol Biochem. 2001;39(12):1067–73. 10.1016/s0981-9428(01)01335-3

[42] Atkinson NJ, Lilley CJ, Urwin PE. Identification of genes involved in the response of *Arabidopsis* to simultaneous biotic and abiotic stresses. Plant Physiol. 2013;162(4):2028–41. 10.1104/pp.113.222372 23800991 PMC3729780

[43] Ye N, Zhu G, Liu Y, Li Y, Zhang J. ABA controls H_2_O_2_ accumulation through the induction of *OsCATB* in rice leaves under water stress. Plant and Cell Physiol. 2011 10.1093/pcp/pcr028 21398647

[44] Ren H, Wei K, Jia W, Davies WJ, Zhang J. Modulation of root signals in relation to stomatal sensitivity to root-sourced abscisic acid in drought-affected plants. J Integr Plant Biol. 2007;49(10):1410–20. 10.1111/j.1672-9072.2007.00549.x

[45] Shi Y, Tian S, Hou L, Huang X, Zhang X, Guo H, et al Ethylene signaling negatively regulates freezing tolerance by repressing expression of CBF and type-A ARR genes in *Arabidopsis* . Plant Cell. 2012;24(6):2578–95. 10.1105/tpc.112.098640 22706288 PMC3406918

[46] Usadel B, BlÄSing OE, Gibon Y, Poree F, HÖHne M, GÜNter M, et al Multilevel genomic analysis of the response of transcripts, enzyme activities and metabolites in *Arabidopsis* rosettes to a progressive decrease of temperature in the non-freezing range. Plant, Cell Environ. 2008;31(4):518–47. 10.1111/j.1365-3040.2007.01763.x 18088337

[47] Cook D, Fowler S, Fiehn O, Thomashow MF. A prominent role for the CBF cold response pathway in configuring the low-temperature metabolome of Arabidopsis. Proc Natl Acad Sci USA. 2004;101(42):15243–8. 10.1073/pnas.0406069101 15383661 PMC524070

[48] Alcázar R, Marco F, Cuevas J, Patron M, Ferrando A, Carrasco P, et al Involvement of polyamines in plant response to abiotic stress. Biotechnology Letters. 2006;28(23):1867–76. 10.1007/s10529-006-9179-3 17028780

[49] Reaney MJT, Gusta LV, Abrams SR, Robertson AJ. The effects of abscisic acid, kinetin, and gibberellin on freezing tolerance in smooth bromegrass (Bromus inermis) cell suspensions. Can J Bot. 1989;67(12):3640–6. 10.1139/b89-445

[50] Tran LSP, Urao T, Qin F, Maruyama K, Kakimoto T, Shinozaki K, et al Functional analysis of AHK1/ATHK1 and cytokinin receptor histidine kinases in response to abscisic acid, drought, and salt stress in Arabidopsis. Proc Natl Acad Sci. 2007;104(51):20623–8. 10.1073/pnas.0706547105 18077346 PMC2154481

[51] Matsuda O, Sakamoto H, Nakao Y, Oda K, Iba K. CTD phosphatases in the attenuation of wound-induced transcription of jasmonic acid biosynthetic genes in Arabidopsis. Plant J. 2009;57(1):96–108. 10.1111/j.1365-313X.2008.03663.x 18764923

[52] Gehring CA, Irving HR, McConchie R, Parish RW. Jasmonates induce intracellular alkalinization and closure of *Paphiopedilum* guard cells. Ann Bot. 1997;80(4):485–9. 10.1006/anbo.1997.0471

[53] Suhita D, Raghavendra AS, Kwak JM, Vavasseur A. Cytoplasmic alkalization precedes reactive oxygen species production during methyl jasmonate- and abscisic acid-Induced stomatal closure. Plant Physiol. 2004;134(4):1536–45. 10.1104/pp.103.032250 15064385 PMC419829

[54] Hossain MA, Munemasa S, Uraji M, Nakamura Y, Mori IC, Murata Y. Involvement of edogenous abscisic acid in methyl jasmonate-induced stomatal closure in *Arabidopsis* . Plant Physiol. 2011;156(1):430–8. 10.1104/pp.111.172254 21402795 PMC3091061

[55] Wyatt SE, Rashotte AM, Shipp MJ, Robertson D, Muday GK. Mutations in the gravity persistence signal loci in Arabidopsis disrupt the perception and/or signal transduction of gravitropic stimuli. Plant Physiol. 2002;130(3):1426–35. 10.1104/pp.102.010579 12428007 PMC166661

[56] Shibasaki K, Uemura M, Tsurumi S, Rahman A. Auxin response in *Arabidopsis* under cold stress: underlying molecular mechanisms. Plant Cell. 2009;21(12):3823–38. 10.1105/tpc.109.069906 20040541 PMC2814496

[57] Nakashima K, Tran L-SP, Van Nguyen D, Fujita M, Maruyama K, Todaka D, et al Functional analysis of a NAC-type transcription factor OsNAC6 involved in abiotic and biotic stress-responsive gene expression in rice. Plant J. 2007;51(4):617–30. 10.1111/j.1365-313X.2007.03168.x 17587305

[58] Nuruzzaman M, Manimekalai R, Sharoni AM, Satoh K, Kondoh H, Ooka H, et al Genome-wide analysis of NAC transcription factor family in rice. Gene. 2010;465(1–2):30–44. 10.1016/j.gene.2010.06.008 20600702

[59] Figueiredo DD, Barros PM, Cordeiro AM, Serra TS, Lourenço T, Chander S, et al Seven zinc-finger transcription factors are novel regulators of the stress responsive gene *OsDREB1B* . J Exp Bot. 2012 10.1093/jxb/ers035 22412187

[60] Valverde F. CONSTANS and the evolutionary origin of photoperiodic timing of flowering. J Exp Bot. 2011 10.1093/jxb/erq449 21239381

[61] Chen J, Chen J-y, Wang J-n, Kuang J-f, Shan W, Lu W-j. Molecular characterization and expression profiles of *MaCOL1*, a *CONSTANS*-like gene in banana fruit. Gene. 2012;496(2):110–7. 10.1016/j.gene.2012.01.008 22285923

[62] Fowler SG, Cook D, Thomashow MF. Low temperature induction of Arabidopsis *CBF1*, *2*, and *3* is gated by the circadian clock. Plant Physiol. 2005;137(3):961–8. 10.1104/pp.104.058354 15728337 PMC1065397

[63] Yang A, Dai X, Zhang WH. A R2R3-type MYB gene, *OsMYB2*, is involved in salt, cold, and dehydration tolerance in rice. J Exp Bot. 2012 10.1093/jxb/err431 PMC3346221 22301384

[64] Wang Y, Guo H, Li H, Zhang H, Miao X. Identification of transcription factors potential related to brown planthopper resistance in rice via microarray expression profiling. BMC Genomics. 2012;13(1):1–12. 10.1186/1471-2164-13-687 23228240 PMC3538557

[65] D'Agostino IB, Deruère J, Kieber JJ. Characterization of the response of the *Arabidopsis* response regulator gene family to cytokinin. Plant Physiol. 2000;124(4):1706–17. 10.1104/pp.124.4.1706 11115887 PMC59868

[66] Woeste KE, Vogel JP, Kieber JJ. Factors regulating ethylene biosynthesis in etiolated Arabidopsis thaliana seedlings. Physiol Plant. 1999;105(3):478–84. 10.1034/j.1399-3054.1999.105312.x

[67] Vogel JP, Woeste KE, Theologis A, Kieber JJ. Recessive and dominant mutations in the ethylene biosynthetic gene ACS5 of *Arabidopsis* confer cytokinin insensitivity and ethylene overproduction, respectively. Proceedings of the National Academy of Sciences of the United States of America. 1998;95(8):4766–71. PMC22565. 9539813 10.1073/pnas.95.8.4766 PMC22565

