# Supplementary figures and images for: Comparative Transcriptome Analysis of Shoots and Roots of TNG67 and TCN1 Rice Seedlings under Cold Stress and Following Subsequent Recovery: Insights into Metabolic Pathways, Phytohormones, and Transcription Factors
Source: PLoS One. 2015 Jul 2;10(7):e0131391. doi: 10.1371/journal.pone.0131391 (PMC4489882; doi:10.1371/journal.pone.0131391)

# Correlation between Microarray and qPCR

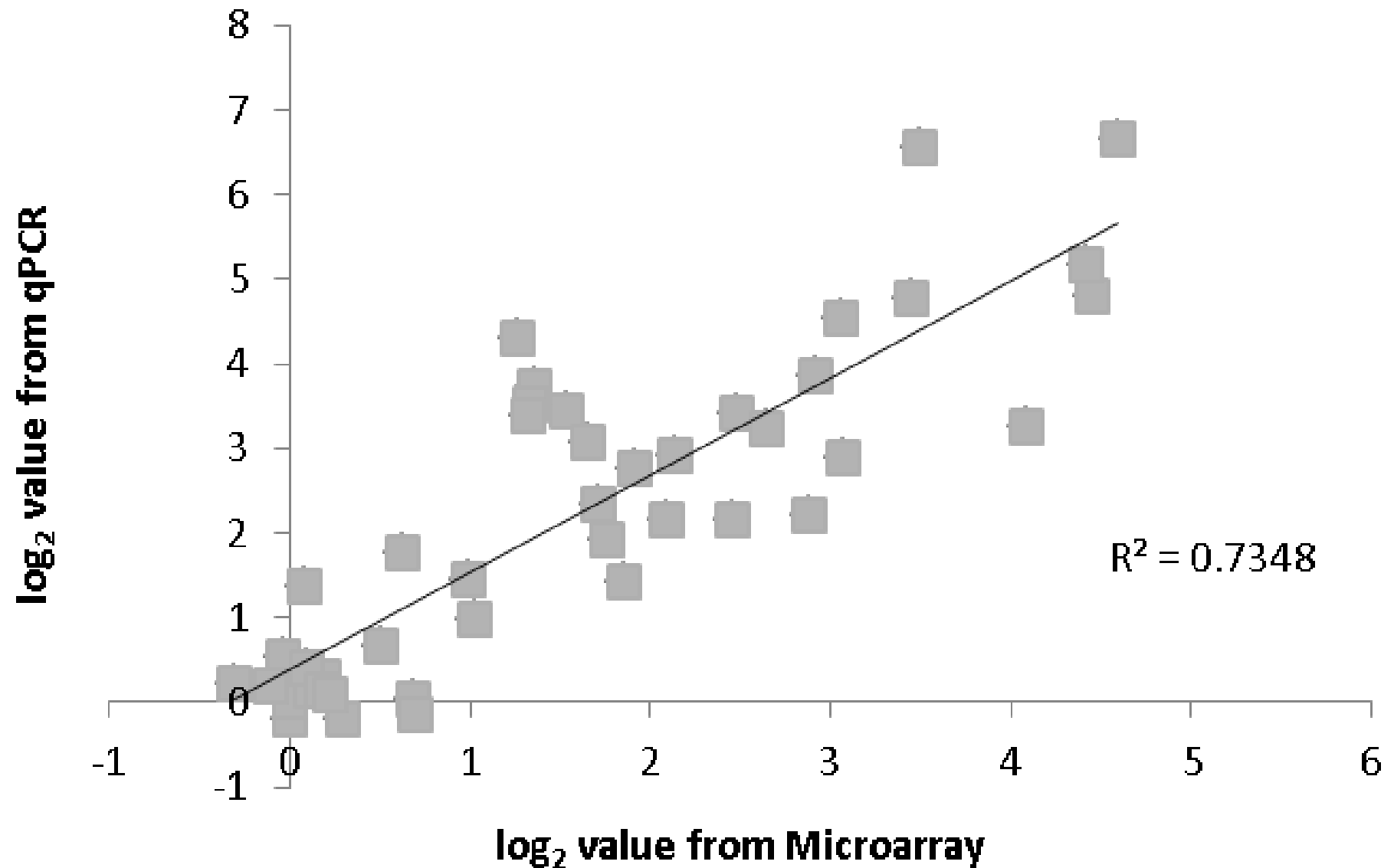

Supplement: S1 Fig — (PDF) [file pone.0131391.s001.pdf]

(A)3/0 hr cold treatment

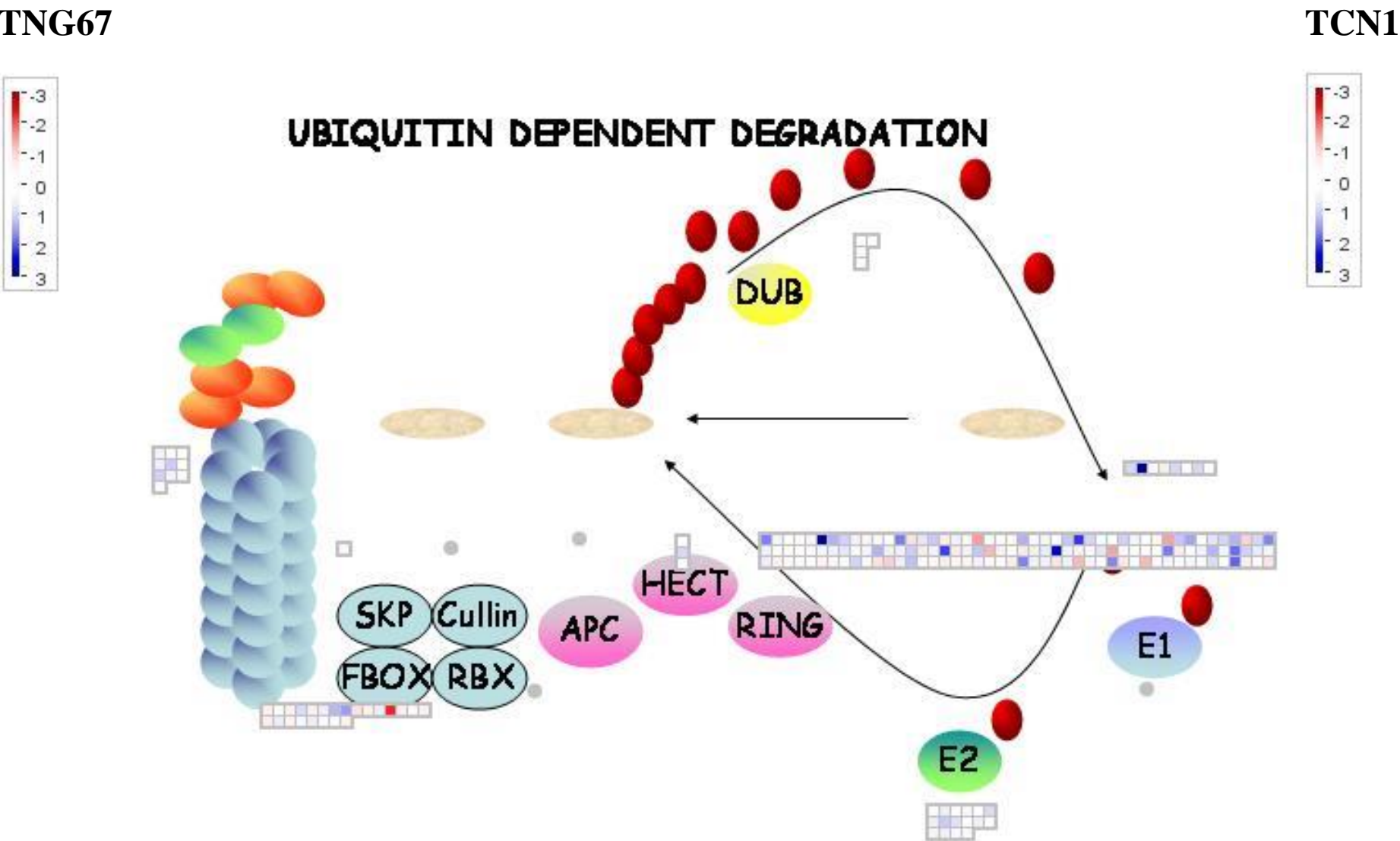

(B)24/0 hr cold treatment

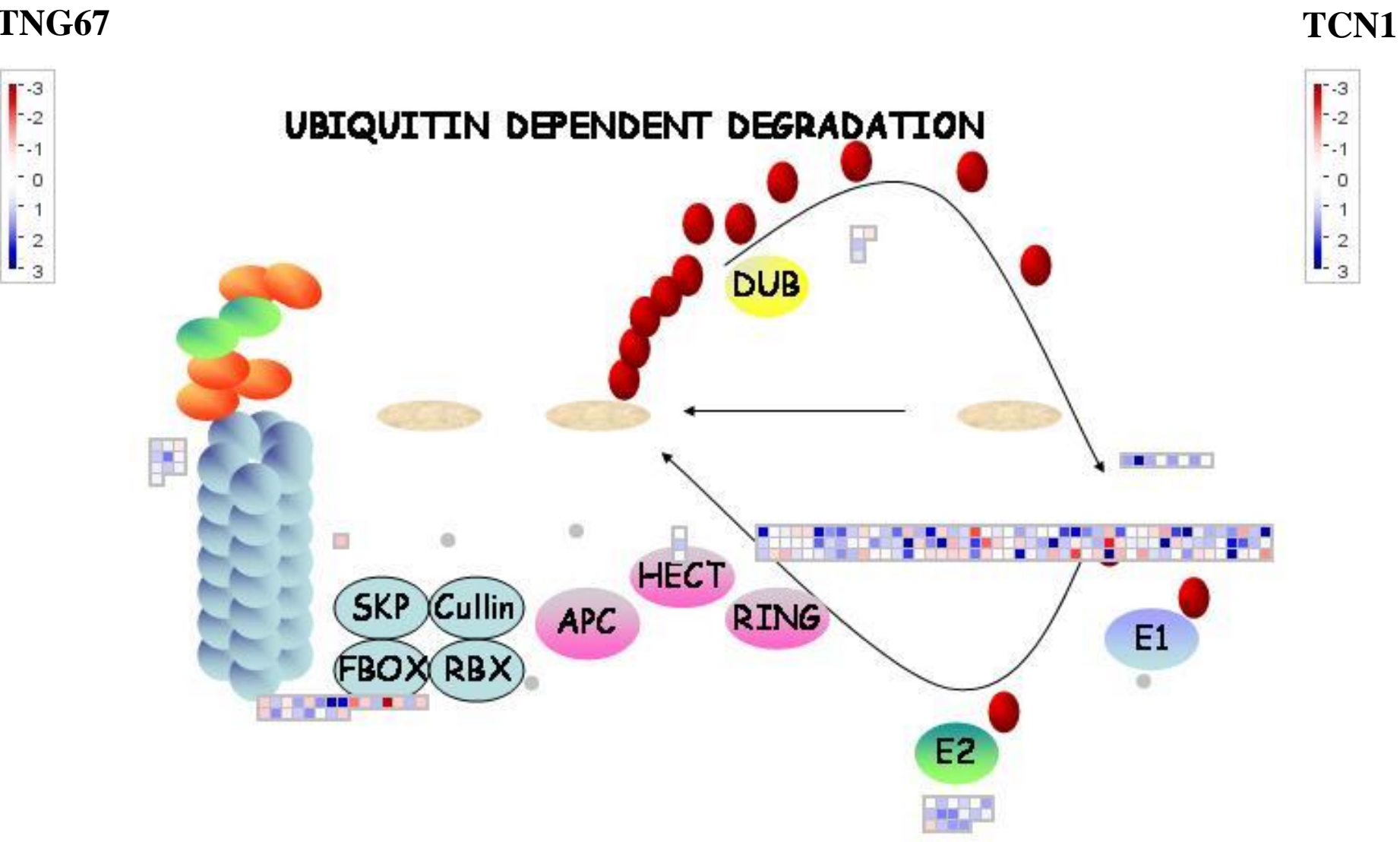

(C)24re/0 hr cold treatment

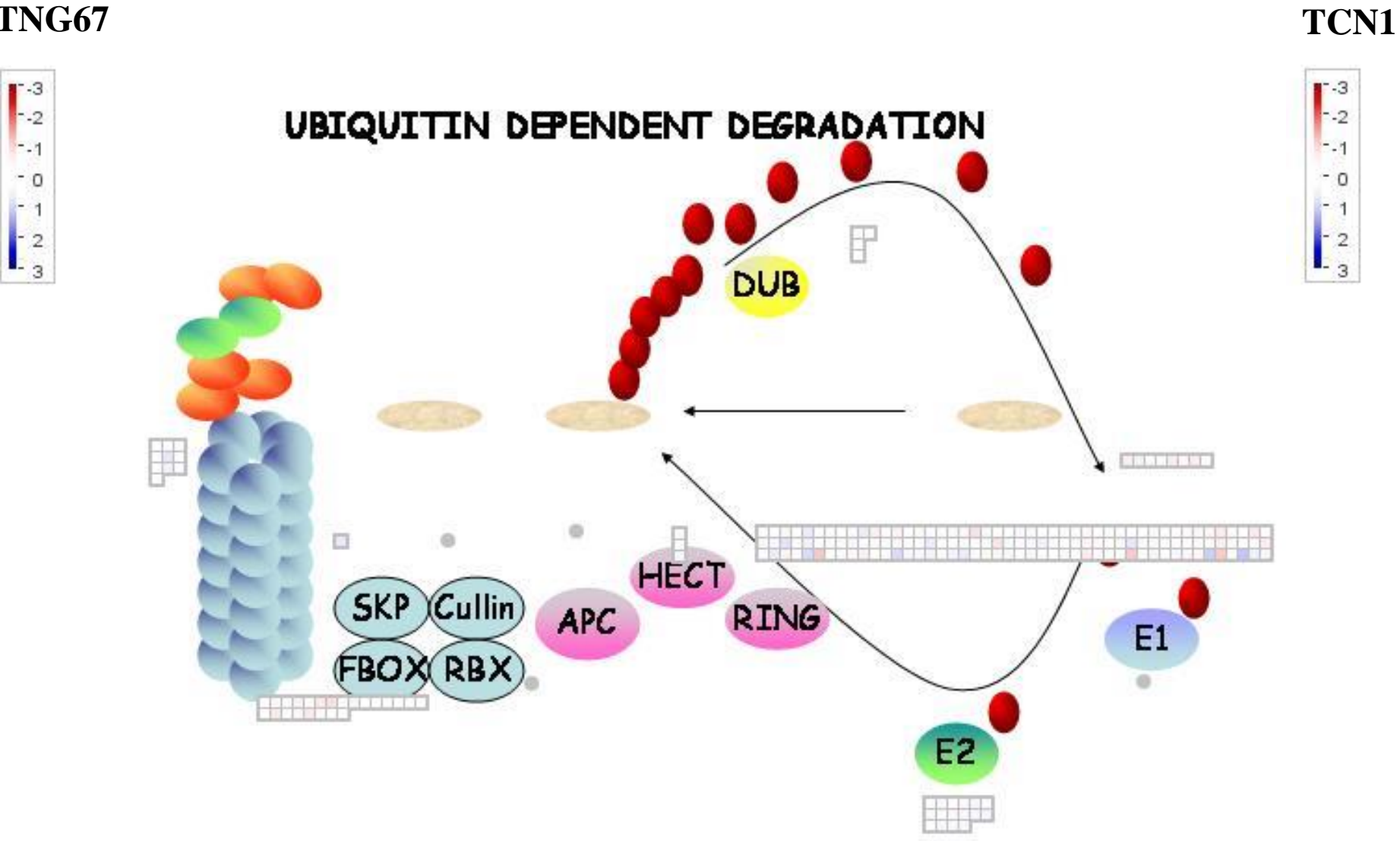

Supplement: S2 Fig — (PDF) [file pone.0131391.s002.pdf]

**Control**

**Cold stress**

**Control**

**Recovery**

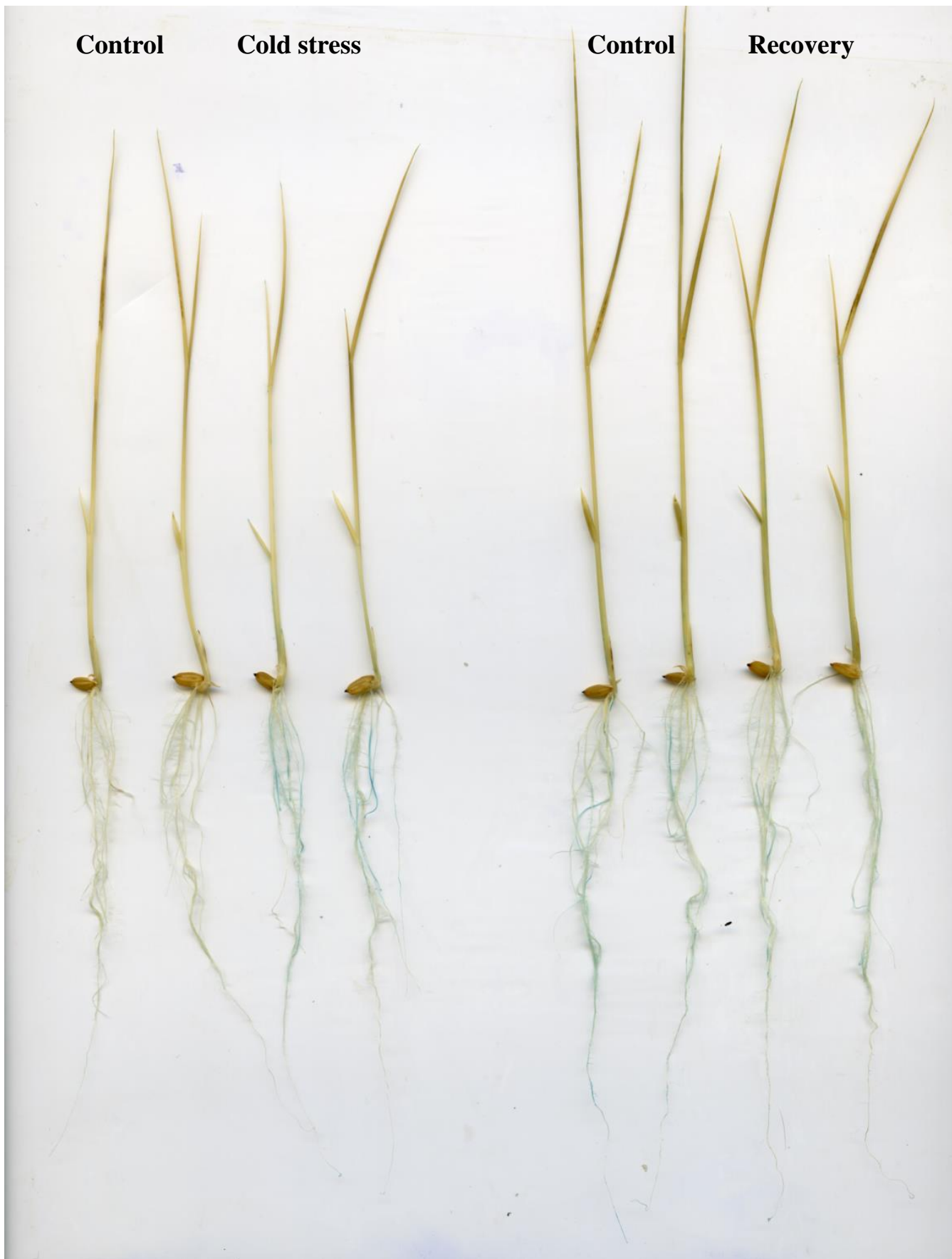

Supplement: S3 Fig — (PDF) [file pone.0131391.s003.pdf]

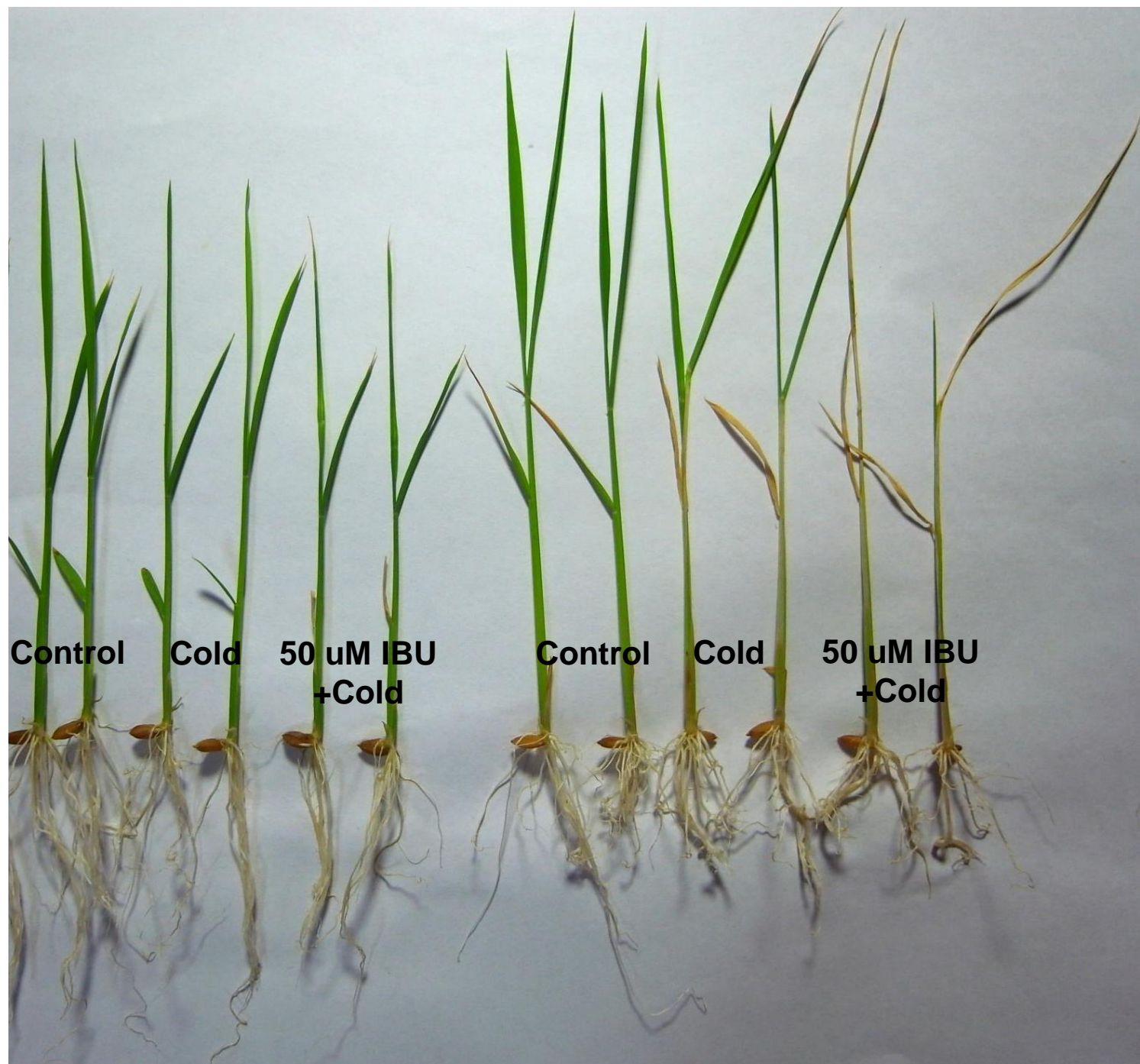

Supplement: S4 Fig — (PDF) [file pone.0131391.s004.pdf]

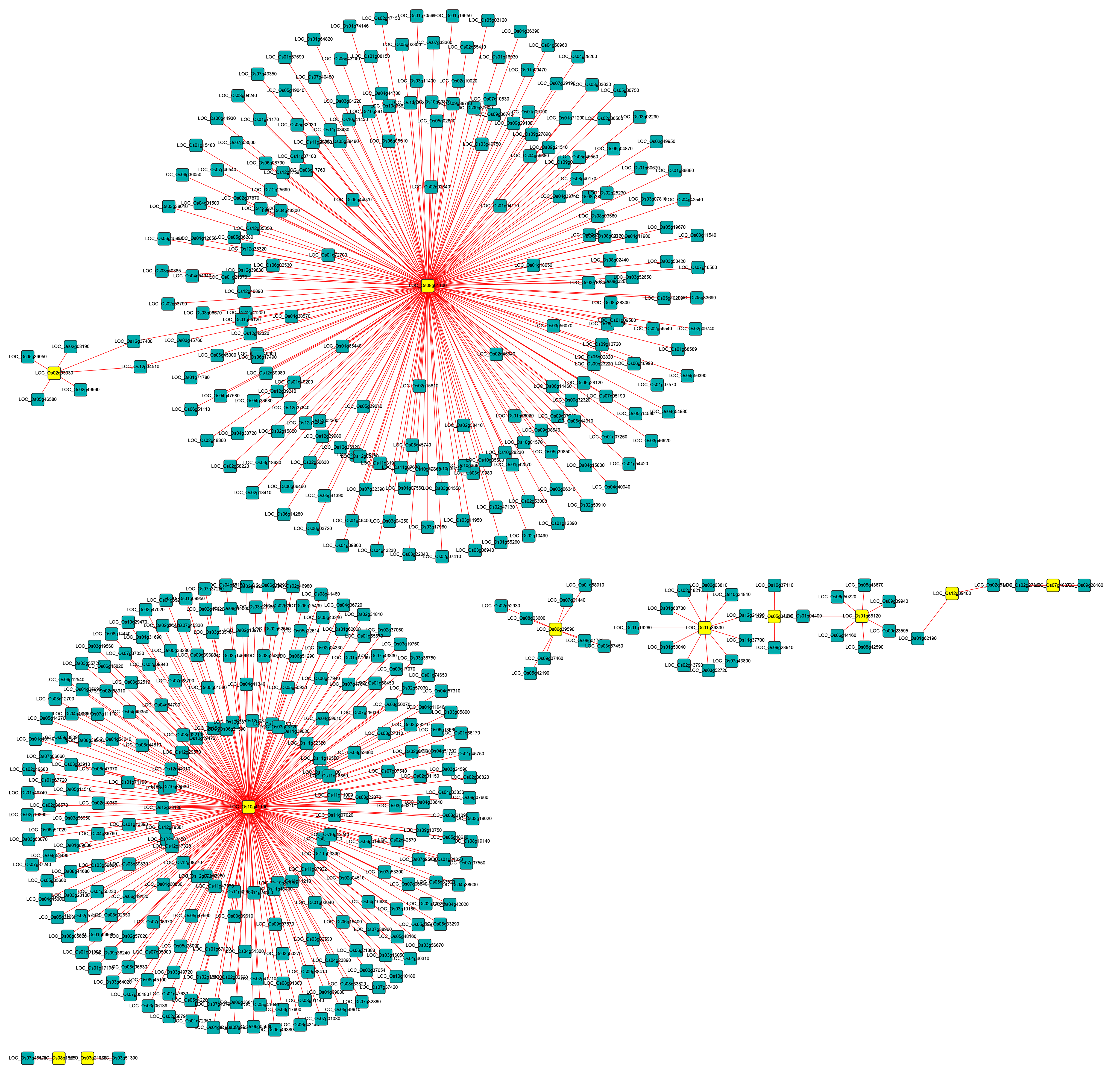

Supplement: S5 Fig — (TIF) [file pone.0131391.s005.tif]

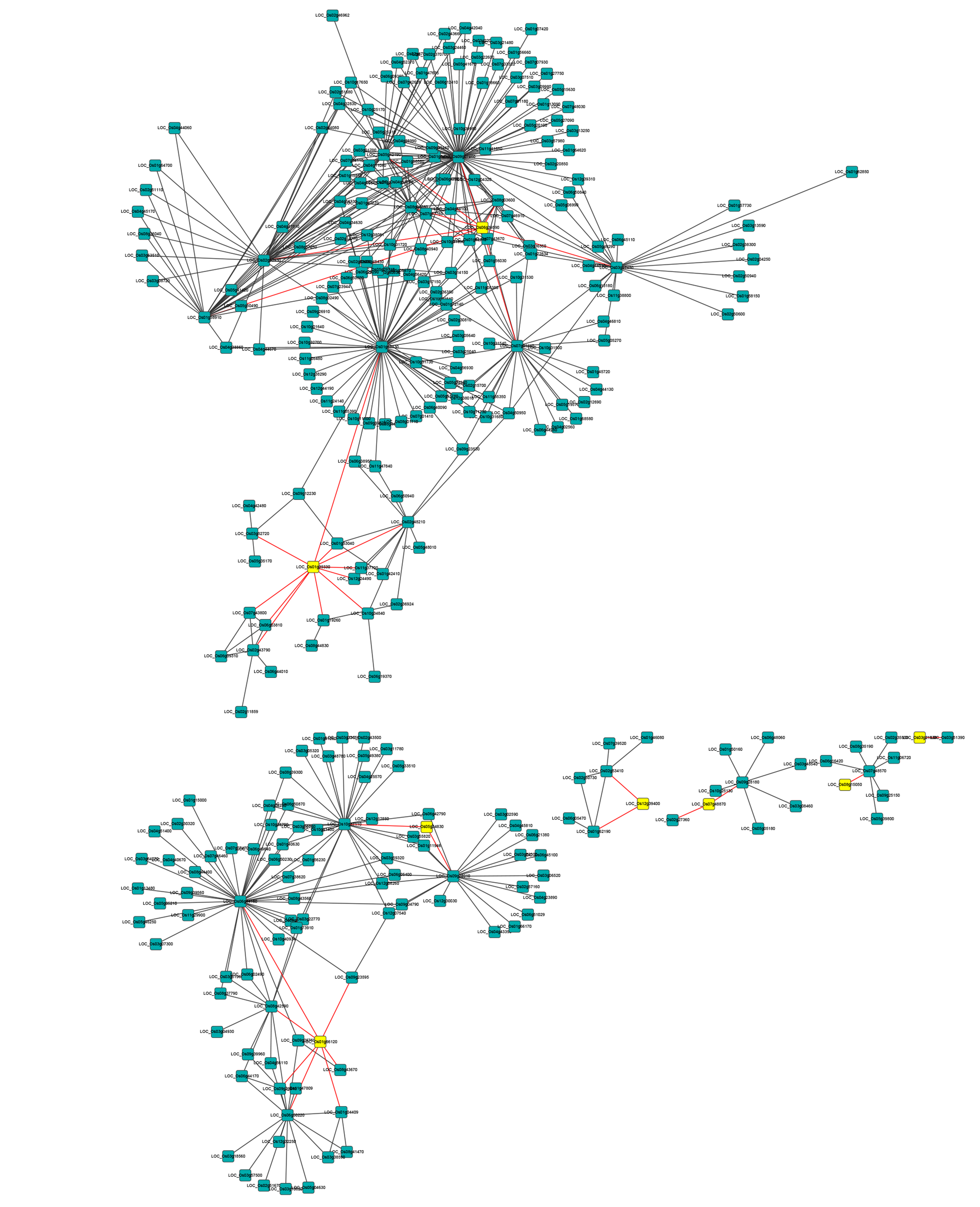

Supplement: S6 Fig — (TIF) [file pone.0131391.s006.tif]

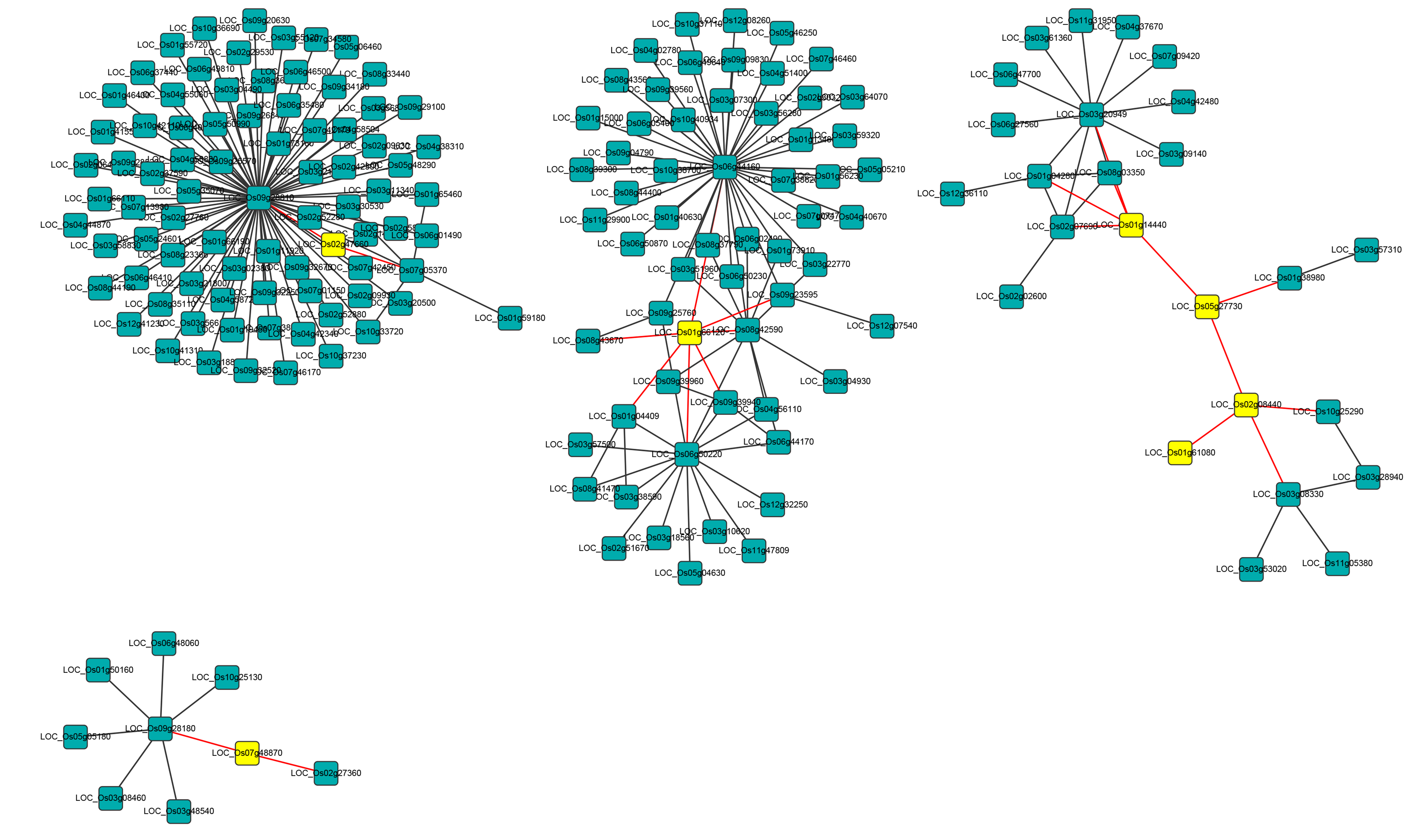

Supplement: S7 Fig — (TIF) [file pone.0131391.s007.tif]

Portion of TFs gene expression affected by phytohormones

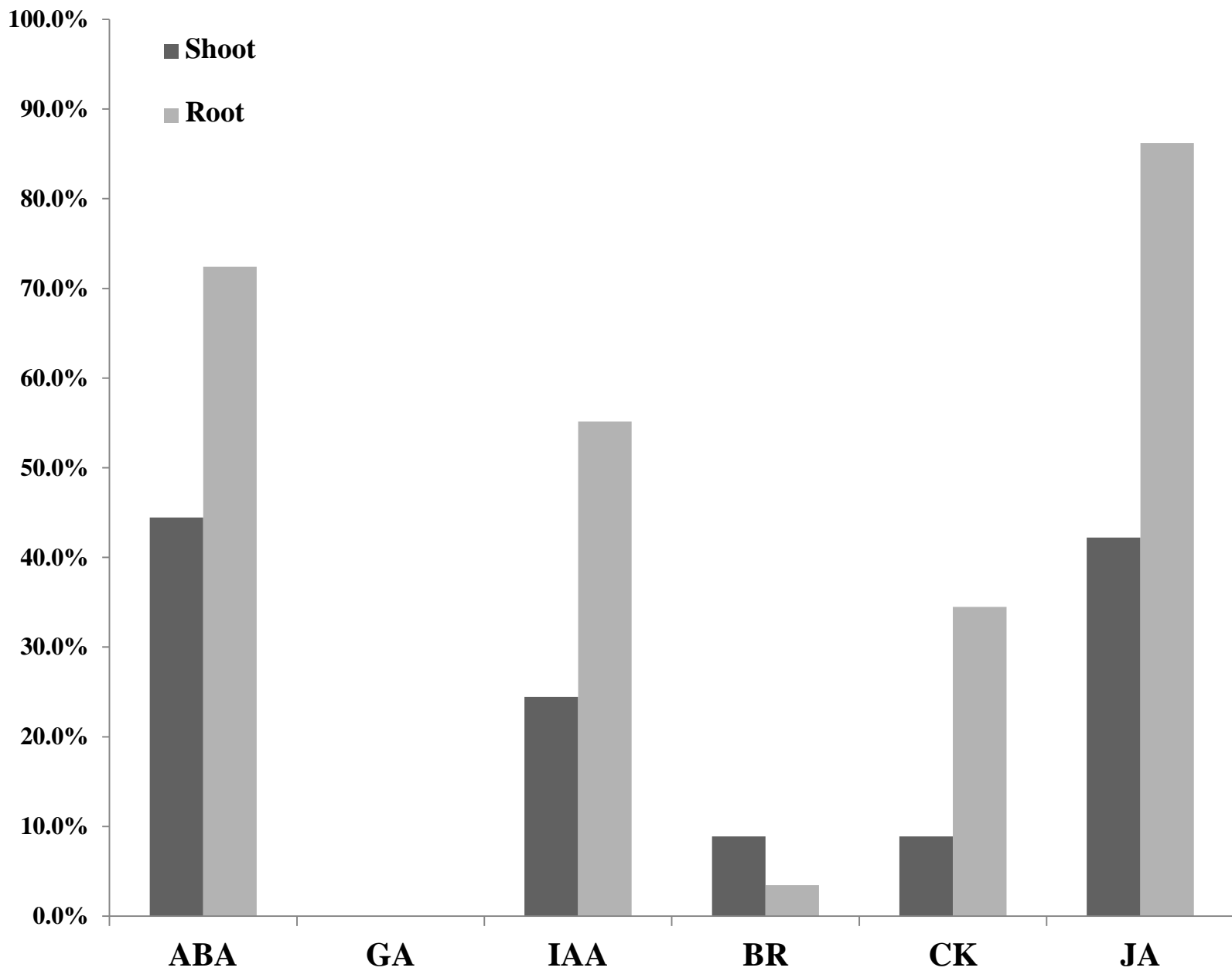

Supplement: S8 Fig — (PDF) [file pone.0131391.s008.pdf]
