# Supplementary material for: Comparative Transcriptome Analysis of Shoots and Roots of TNG67 and TCN1 Rice Seedlings under Cold Stress and Following Subsequent Recovery: Insights into Metabolic Pathways, Phytohormones, and Transcription Factors
Source: PLoS One. 2015 Jul 2;10(7):e0131391. doi: 10.1371/journal.pone.0131391 (PMC4489882; doi:10.1371/journal.pone.0131391)
Supplement: S1 Table — (PDF) [file pone.0131391.s009.pdf]

MSU_Gene_ID	MSU_Description	Primer	
LOC_Os01g06590	zinc finger, C3HC4 type domain containing protein, expressed	F	GCGAGGACAAACAACAATATGG
		R	AAAGGATATCAGCTATGGTAACACACAA
LOC_Os01g22490	40S ribosomal protein S27a, putative, expressed	F	GCGGAAGTAAGGAAGGAGGAGGA
		R	GGTGATGCTAAGGTGTTCAGTTCCA
LOC_Os01g41420	transmembrane amino acid transporter protein, putative, expressed	F	TGGTTGCTGTTGGTCTCTGATAGTA
		R	TTTTTTCAAGCGCCCTTTATTT
LOC_Os01g51690	OsWRKY59 - Superfamily of TFs having WRKY and zinc finger domains, expressed	F	ATTAGCTAGGTTTGCTCACTTTTGG
		R	GACATTTTTTTGGGCGTTTTG
LOC_Os01g55240	gibberellin 2-beta-dioxygenase, putative, expressed	F	TGATTTTCTCGTCATGCTTTCTG
		R	AACACAAGCCACGTACTATACACACA
LOC_Os01g74020	MYB family transcription factor, putative, expressed	F	GCTGTTTGTTTGTAGCTGTTGTTAATT
		R	CAAGGGAACCATACATATGCATGA
LOC_Os02g47470	cytochrome P450, putative, expressed	F	GGAGACCGAACGGTGGATT
		R	CCTCTGCTCCTCCTCTCGATT
LOC_Os03g08310	ZIM domain containing protein, putative, expressed	F	AATGGCCTTGAGTCGGTAGATC
		R	CCCCATAGGAATCGAAAAAAAA
LOC_Os04g42090	CPuORF7 - conserved peptide uORF-containing transcript, expressed	F	TTCTATAGCTTTCCCCGTGTTCAT
		R	GTCCATGCCAAGGCGAAT
LOC_Os05g05680	1-aminocyclopropane-1-carboxylate oxidase, putative, expressed	F	CATAACCGCTTTTGCTATTCAAGA
		R	CAAACCGTGACACACTAAAATACCA

LOC_Os06g11170	formin-binding protein-related, putative, expressed	F	TGGATAAATTCCCTTTCCTGAAAC
		R	GTGTATCCAGTGTGAAAGAGCAAAA
LOC_Os06g40170	phospholipase D, putative, expressed	F	AAGTTTGTGCTGGTGGTGCAT
		R	CGTTGAAAAACAAACACATTCTGAT
LOC_Os06g40180	phospholipase D, putative, expressed	F	TGTGATCGAGTTGCCATTCCT
		R	TCAACCGGAGATAGATTACATTGC
LOC_Os08g04800	triacylglycerol lipase like protein, putative, expressed	F	TGCACTGACGGCGACATG
		R	CCATCAGGAGGAAACAAATTAACA
LOC_Os08g33620	arginine decarboxylase, putative|pyridoxal-dependent decarboxylase protein, putative, expressed	F	GGCAATCGGGCAGATGTTC
		R	TGCCTCCCGCTGATGAAG
LOC_Os09g27820	1-aminocyclopropane-1-carboxylate oxidase protein, putative, expressed	F	TCGATCCGTGTCATAGCAAGTC
		R	AATAATGCATCCAAACCAAGATACAG
LOC_Os09g35030	1-aminocyclopropane-1-carboxylate oxidase protein, putative, expressed	F	GCATGGGTTGTAGGTTCGATAAA
		R	TCCTGGTGACCCTTACTTTTCAC
LOC_Os10g25230	ZIM domain containing protein, putative, expressed	F	GCACGATTATTCCTATCTGTCACAA
		R	TCCATCATCGTTCTTTCAGTATTTTT
LOC_Os12g39630	CAMK_CAMK_like.49 - CAMK includes calcium/calmodulin depedent protein kinases, expressed	F	AGAGCAGCGGAGATCGTATATG
		R	TTCTGGAGCTCTACCTGCTCTGA
**Transcription factor**			
LOC_Os08g01100	HMGB	F	ACCCAAGAAAGGCACTGATGA
		R	ATTGGACCGGTGGCCTAGA
LOC_Os10g41100	CO-like	F	GGCTCGACCTCGGATGGT

		R	CATGCTTGGCCTATGTACGTAGAGT
LOC_Os02g03030	PHD	F	CGGAAGAAGCAGAATCTCAAGTG
		R	ACAGCCTCTCCCCTTTGGA
LOC_Os06g39590	OsIAA23	F	TGGTTCGGTTTCCTGTGACA
		R	GAACAAAGTCAACAACAGCAACAA
LOC_Os01g39330	bHLH	F	CAGCTTCCACACGCTCAGATC
		R	GATCTCCGTCTCCAGCGACTT
LOC_Os01g66120	OsSNAC2	F	ATGGGAGAGACGGGTGGATAT
		R	TGAACCCTCAAAGACAAAACTACGT
LOC_Os05g34830	NAC	F	CCCGAGATGCGTGTAGGAA
		R	GAGAGAAATACGCCCAGATCGA
LOC_Os02g47660	NAC	F	TGCTCGACGAGATCATCAACTAC
		R	GAAGTTCACCACCGGGTTTG
LOC_Os07g48870	MYB	F	CATGGATGGAGATAAACTACAGTGCTA
		R	CGTCCCGAGAAATTAAGAATGG
LOC_Os01g14440	OsWRKY 1v2	F	CAACAGCGAGACGCAGTAAAAA
		R	AACCAAAGGAGAGAAATCAAGAACCTA
LOC_Os05g27730	OsWRKY53	F	ACACGGCAATACACACATACATACTC
		R	ACAAATTGGATCTCACTCCCTAGCT
LOC_Os02g08440	OsWRKY71	F	CAAGGATTGACGATTGCTACTGA
		R	GAGAGGCAGAGACAGGAGAGGAT
LOC_Os01g61080	OsWRKY24	F	AGCAGCAGAGGCAGAACGA

		R	TTCCTACGACGACGATTCTTTCA
