# Supplementary material for: Comparative Transcriptome Analysis of Shoots and Roots of TNG67 and TCN1 Rice Seedlings under Cold Stress and Following Subsequent Recovery: Insights into Metabolic Pathways, Phytohormones, and Transcription Factors
Source: PLoS One. 2015 Jul 2;10(7):e0131391. doi: 10.1371/journal.pone.0131391 (PMC4489882; doi:10.1371/journal.pone.0131391)
Supplement: S9 Table — A heat map of TF-encoding DEGs constructed based on the microarray data is shown. In this table, “S” represents shoot and “R” represents root. Genes that were induced or repressed by a given hormone treatment listed in RiceXPro database are denoted by “↑” and “↓”, respectively. “N” indicates that no data are available in RiceXPro database for these TFs. “-” indicates no effect in the presence of specific plant hormones. (PDF) [file pone.0131391.s017.pdf]

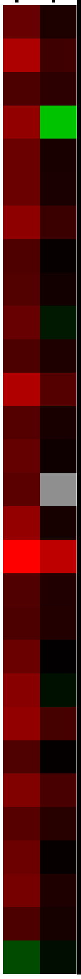

MUS_Gene_Symbol		ABA		GA		IAA		BR		CK		JA	
		S	R	S	R	S	R	S	R	S	R	S	R
LOC_Os01g64790	AP2/ERF	↓	↑	–	–	↑	↑	–	–	–	↑	↑	↑
LOC_Os06g07030	AP2/ERF	↑	↑	–	–	↑	–	–	–	–	–	↑	↑
LOC_Os09g11480	AP2/ERF	–	↓	–	–	–	–	–	–	–	–	↓	–
LOC_Os09g28440	AP2/ERF	–	–	–	–	–	–	–	–	–	–	–	↑
LOC_Os09g39850	AP2/ERF	–	–	–	–	↑	↑	–	–	–	–	↓	↑
LOC_Os10g35300	AP2/ERF	↑	↑	–	–	↑	–	–	–	–	–	↑	–
LOC_Os03g60560	C2H2	↑	↑	–	–	–	↑	–	–	–	–	–	↑
LOC_Os03g60570	C2H2	↑	↑	–	–	–	↑	–	–	–	–	–	↑
LOC_Os04g35800	C3H	–	–	–	–	–	–	–	–	–	–	–	↓
LOC_Os06g16370	CO-like	–	↑	–	–	–	–	–	–	–	–	–	↑
LOC_Os02g43150	GATA	–	↑	–	–	–	–	–	–	–	–	–	–
LOC_Os01g53220	HSF	↑	↑	–	–	–	–	–	–	–	↑	–	↓
LOC_Os03g20090	MYB	↑	–	–	–	–	–	–	–	–	↓	–	↑
LOC_Os07g48870	MYB	↑	↑	–	–	–	↑	–	–	↓	–	↑	↑
LOC_Os11g45740	MYB	↑	↑	–	–	–	↑	–	–	↓	↓	↑	↑
LOC_Os01g60020	NAC	–	↑	–	–	↑	↑	↑	–	↓	↑	↑	↑
LOC_Os01g66120	NAC	↑	↑	–	–	–	↑	–	–	–	↓	↑	↑
LOC_Os11g03370	NAC	–	–	–	–	–	–	–	↑	–	–	↑	↑
LOC_Os01g14440	WRKY	↑	↑	–	–	↑	↑	–	–	–	–	↑	↑
LOC_Os01g51690	WRKY	–	–	–	–	↑	–	↑	–	–	–	–	↑
LOC_Os01g61080	WRKY	–	–	–	–	↑	↑	–	–	–	–	–	↑
LOC_Os02g08440	WRKY	↑	↑	–	–	↑	↑	–	–	–	↑	↑	↑
LOC_Os03g21710	WRKY	–	↓	↓	–	–	↑	–	–	–	–	–	–
LOC_Os04g21950	WRKY	–	↑	–	–	–	–	–	–	–	–	–	↑
LOC_Os05g27730	WRKY	–	↑	–	–	↑	↑	–	–	–	↑	↑	↑
LOC_Os05g46020	WRKY	↑	↑	–	–	↑	↑	–	–	–	↑	↑	↑
LOC_Os08g29660	WRKY	–	↓	–	–	–	↑	–	–	–	–	–	↑
LOC_Os12g02440	WRKY	–	–	–	–	–	–	–	–	–	–	↑	↑
LOC_Os02g47660	bHLH	↑	↑	–	–	↑	↑	↑	–	–	↑	–	↓
