# Supplementary material for: Comparative Transcriptome Analysis of Shoots and Roots of TNG67 and TCN1 Rice Seedlings under Cold Stress and Following Subsequent Recovery: Insights into Metabolic Pathways, Phytohormones, and Transcription Factors
Source: PLoS One. 2015 Jul 2;10(7):e0131391. doi: 10.1371/journal.pone.0131391 (PMC4489882; doi:10.1371/journal.pone.0131391)
Supplement: S10 Table — A heat map of TF-encoding DEGs constructed based on the microarray data is shown. In this table, “S” represents shoot and “R” indicates root. Genes that were induced or repressed by a given hormone treatment listed in RiceXPro database are denoted by “↑” and “↓”, respectively. “N” indicates that no data are available in RiceXPro database for these TFs. “-” indicates no effect in the presence of specific plant hormones. (PDF) [file pone.0131391.s018.pdf]

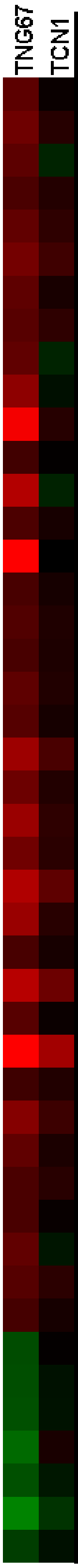

MUS_Gene_Symbol	Hormone	ABA		GA		IAA		BR		CK		JA	
		S	R	S	R	S	R	S	R	S	R	S	R
LOC_Os02g42585	AP2/ERF	N	N	N	N	N	N	N	N	N	N	N	N
LOC_Os02g54050	AP2/ERF	N	N	N	N	N	N	N	N	N	N	N	N
LOC_Os04g34970	AP2/ERF	N	N	N	N	N	N	N	N	N	N	N	N
LOC_Os04g57340	AP2/ERF	–	↑	–	–	–	–	–	–	–	–	↑	↑
LOC_Os08g31580	AP2/ERF	↑	↑	–	–	↑	↑	↑	–	–	↑	↑	↓
LOC_Os05g48590	AUX/IAA	–	–	–	–	–	↑	–	–	–	–	–	↑
LOC_Os06g39590	AUX/IAA	–	↓	–	–	–	↑	–	–	–	↑	–	↑
LOC_Os01g39330	bHLH	–	↓	–	–	↑	↑	–	–	–	↑	↑	↑
LOC_Os03g55550	bHLH	N	N	N	N	N	N	N	N	N	N	N	N
LOC_Os09g25430	C2H2	–	–	–	–	–	–	–	–	–	–	–	↓
LOC_Os11g47630	C2H2	–	–	–	–	–	–	–	–	↑	↑	↑	–
LOC_Os12g39400	C2H2	↑	↑	–	–	–	–	–	–	–	–	↑	↑
LOC_Os06g16370	CO-like	–	↑	–	–	–	–	–	–	–	–	–	↑
LOC_Os08g15050	CO-like	↑	↑	–	–	–	–	–	–	–	↓	–	–
LOC_Os10g41100	CO-like	–	–	–	–	–	↓	–	↑	–	↑	–	–
LOC_Os02g43170	DBB	↓	–	–	–	↓	–	–	–	–	↑	–	↑
LOC_Os08g38220	Dof	–	–	–	–	–	–	–	–	–	–	–	↓
LOC_Os04g33950	E2F/DP	–	↑	–	–	–	–	–	–	–	–	–	↑
LOC_Os07g39470	GRAS	–	↑	–	–	–	–	–	–	–	↑	–	↑
LOC_Os01g53220	HSF	↑	↑	–	–	–	–	–	–	–	↑	–	↓
LOC_Os02g40530	MYB	↑	↑	–	–	–	–	–	–	↓	↑	↑	↑
LOC_Os07g48870	MYB	↑	↑	–	–	–	↑	–	–	↓	–	↑	↑
LOC_Os10g33810	MYB	↑	↓	–	–	↑	–	–	–	–	–	↑	↑
LOC_Os01g41900	MYB_related	–	↓	–	–	–	–	–	–	–	↑	–	↑
LOC_Os05g07010	MYB_related	–	–	–	–	–	–	–	–	–	–	↑	↑
LOC_Os01g15640	NAC	↑	↑	–	–	–	↑	–	–	–	–	–	–
LOC_Os01g66120	NAC	↑	↑	–	–	–	↑	–	–	–	↓	↑	↑
LOC_Os03g21030	NAC	↑	↑	–	–	–	–	–	–	–	–	↑	↑
LOC_Os05g34830	NAC	↑	↑	–	–	↑	↑	–	–	–	↑	–	↑
LOC_Os07g12340	NAC	↑	↑	–	–	↑	↑	–	–	–	–	↑	↑
LOC_Os08g06140	NAC	–	–	–	–	–	–	–	–	–	–	–	–
LOC_Os11g03370	NAC	–	–	–	–	–	–	–	↑	–	–	↑	↑
LOC_Os03g19020	PHD	–	–	–	–	–	–	–	–	–	–	–	↑
LOC_Os04g50120	PLATZ	↑	↑	–	–	–	–	–	–	–	–	–	↑
LOC_Os01g57240	ULT	↑	–	–	–	↑	–	↑	–	–	–	↑	↑
LOC_Os01g51690	WRKY	–	–	–	–	↑	–	↑	–	–	–	↑	↑
LOC_Os04g21950	WRKY	–	↑	–	–	–	–	–	–	–	–	–	↑
LOC_Os05g03740	Trihelix	–	–	–	–	–	–	–	–	–	–	–	–
LOC_Os08g34360	AP2/ERF	↑	–	–	–	↓	↑	–	–	–	–	–	–
LOC_Os01g70870	C2H2	–	–	–	–	–	–	–	↑	–	↑	–	↑
LOC_Os05g28320	MYB	↓	↓	–	–	–	↓	↓	–	–	↑	–	↓
LOC_Os08g10080	NAC	↓	–	–	–	–	↓	–	–	↑	↑	↓	–
LOC_Os01g08820	PHD	–	↓	–	–	↓	↓	–	–	–	↓	↓	↓
LOC_Os02g03030	PHD	–	–	–	–	–	–	–	–	–	–	–	–
LOC_Os08g01100	HMG	↓	↓	–	–	↓	↓	–	–	–	↓	↓	↓
